# Curcubinoyl flavonoids from wild ginseng adventitious root cultures

**DOI:** 10.1038/s41598-021-91850-8

**Published:** 2021-06-09

**Authors:** Qing Liu, Seon Beom Kim, Yang Hee Jo, Jong Hoon Ahn, Ayman Turk, Da Eun Kim, Bo Yoon Chang, Sung Yeon Kim, Cheol-Seung Jeong, Bang Yeon Hwang, So-Young Park, Mi Kyeong Lee

**Affiliations:** 1 College of Pharmacy, Chungbuk National University, Cheongju, 28160 Republic of Korea; 2 College of Pharmacy, Wonkwang University, Iksan, 54538 Republic of Korea; 3 Department of Horticultural Science, Chungbuk National University, Cheongju, 28644 Republic of Korea

**Keywords:** Chemical biology, Natural products

## Abstract

Wild ginseng (*Panax ginseng*) adventitious root cultures were prepared by elicitation using methyl jasmonate and investigated further to find new secondary metabolites. Chromatographic fractionation of wild ginseng adventitious root cultures led to the isolation of eleven compounds. The chemical structures of isolated compounds were identified as four known flavanone derivatives (**1**–**4**), one new curcubinoyl derivative, jasmogin A (**5**) and six new curcubinoyl-flavanone conjugates, jasmoflagins A-F (**6**–**11**) by extensive spectroscopic analysis. Newly isolated curcubinoyl derivatives showed inhibitory activity against lipopolysaccharide-stimulated nitric oxide production in RAW 264.7 macrophages. Therefore, our present study suggested that elicitor stimulated plant cell cultures might contribute to the production of new metabolites.

## Introduction

Natural products contain a variety of ingredients and have long been used to prevent and treat diseases. However, securing natural products is essential in order to develop these natural products, which is sometimes not easy due to various constraints. Plant tissue culture technology is suggested as a powerful tool for obtaining natural substances^1–3^. This is widely used for the production of plant materials because it is less affected by weather and other external conditions than plant cultivation and relatively for a short period of time.

For maximum productivity, culture conditions such as culture medium and the incubation conditions, etc., are optimized when growing plant tissues^4–6^. In particular, the use of elicitors is widely used for increased productivity and useful substances. As elicitors, salicylic acid and methyl jasmonate (MJ), which control the immune of plants, are most widely used^7–8^. These elicitors greatly increase the content of biomass and useful metabolites^4,9^. Moreover, new ingredients have been reported in elicitor-stimulated plant cell culture^10–11^. Therefore, plant tissue culture has become an important tool not only for securing plant materials but also finding new metabolites.

*Panax ginseng* C.A. Meyer (Araliaceae) is commonly known as Korean ginseng. It is one of the most widely used tonic to enhance immune response and consequent health and longevity for over 2000 years in Oriental countries^12^. Various efficacy of *P. ginseng*, including anti-cancer, anti-inflammatory, anti-diabetic, anti-fatigue and neuroprotective activities have been also reported from a lot of research^13–16^.

Ginseng grows in wild environment or is cultivated on farm. Cultivated ginseng is systematically grown on farm under the control of growth condition and harvested after 4–6 year cultivation. The wild ginseng, also called mountain ginseng in Korea, grows without human touch in deep areas with low sunlight and temperature changes. This difference in the cultivation environment and genotypes leads to differences in the composition and efficacy of the two specimens. Wild ginseng has been reported to have enhanced host defense components and biological activities. The concentration of ginsenosides and amino acids in wild ginseng were much higher than those of cultivated ginseng^17,18^.

However, due to the short supply and consequent high price of wild ginseng has limited its usage despite of beneficial biological activities. Therefore, sufficient production is required for the development as products. As a preparation of wild ginseng, tissue culture system is considered as a valuable tool to achieve rapid and stable production of excellent individual. We previously established efficient adventitious root cultures of wild ginseng with fast growth and stable production^19,20^. In addition, we also demonstrated the increased yield and antioxidant activity of MJ-elicitated wild ginseng adventitious root cultures compared to MJ-untreated samples^21^. In the present study, MJ-treated wild ginseng adventitious root cultures were investigated further to find new secondary metabolites.

## Results and discussion

### Isolation of compounds from MJ-treated wild ginseng adventitious root cultures

**Figure 1:**
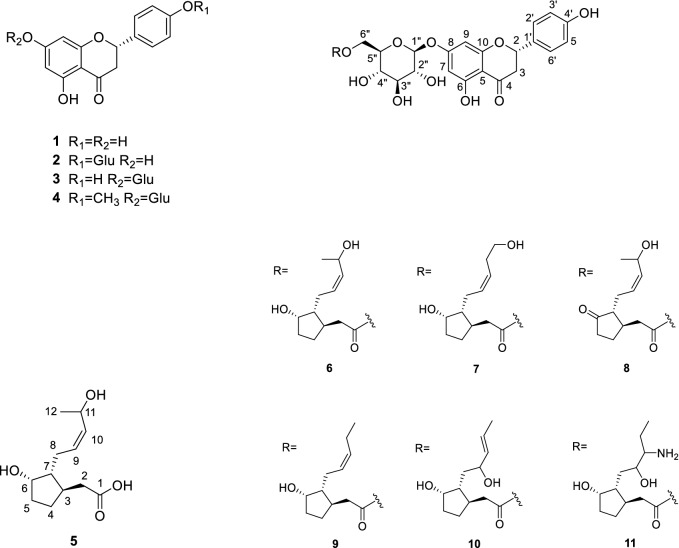
Chemical structures of compounds from MJ-treated wild ginseng adventitious root cultures.

Plant cell cultures were used not only for the stable production but also useful to find new secondary metabolites for better pharmacological activity^9–11^. Investigation on the constituents of the adventitious root cultures of *P. ginseng* yielded eleven compounds including seven new compounds (Fig. 1). Known compounds were identified as naringenin (**1**), naringenin-4′-*O*-*β*-glucoside (**2**), naringenin-7-*O*-*β*-glucoside (**3**) and hesperetin 7-*O*-*β*-glucoside (**4**) by the analysis of their spectroscopic data and comparison with literature values^22–24^.

### Structural determination of the new compounds

**Figure 2:**
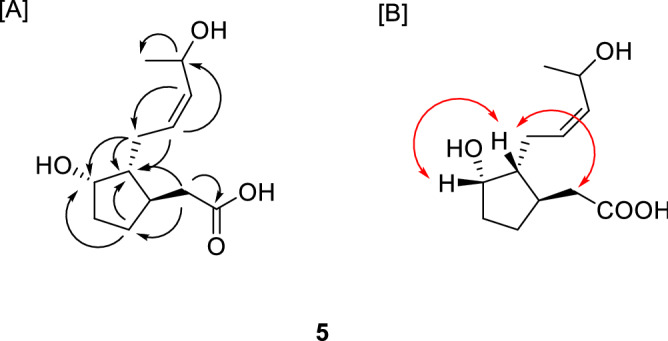
[A] Key HMBC (→) and [B] NOESY (↔) correlations of compound **5.**

Compound **5** was obtained as a white amorphous powder. The molecular formula of **5** was determined as C_12_H_20_O_4_ by its HRESI-MS (*m/z* 251.1254 [M + Na]^+^, calcd. C_12_H_20_NaO_4_ 251.1253). In the ^1^H and ^13^C NMR spectrum, the presence of an olefinic moiety in *cis*-configuration was deduced from the signals at [δ_H_ 5.52 (1H, m, H-9), 5.42 (1H, dd, *J* = 10.4, 8.8 Hz, H-10); δ_C_ 128.8 (C-9), 134.0 (C-10)]. The ^1^H and ^13^C NMR spectrum showed the signals attributed to two hydroxymethines at [δ_H_ 4.15 (1H, m, H-6), 4.68 (1H, dq, *J* = 8.8, 6.4 Hz, H-11)], two methines at [δ_H_ 2.16 (1H, m, H-3), 1.46 (1H, m, H-7)] and one methyl group at [δ_H_ 1.23 (1H, d, *J* = 6.4 Hz, H-12). The ^1^H NMR spectrum and the corresponding carbon signals in HSQC spectrum revealed the presence of four methylenes from the signals at [δ_H_ 2.55 (1H, dd, *J* = 14.4, 3.6 Hz, H-2a), 2.15 (1H, m, H-2b); δ_C_ 39.1 (C-2)], [δ_H_ 1.88 (1H, m, H-4a), 1.64 (1H, m, H-4b); δ_C_ 32.8 (C-4)], [δ_H_ 2.11 (1H, m, H-5a); 1.33 (1H, m, H-5b); δ_C_ 28.8 (C-5)] and [δ_H_ 2.24 (1H, m, H-8a), 2.29 (1H, m, H-8b); δ_C_ 25.5 (C-8)]. In addition, a carbonyl signal was observed at δ_C_ 175.9 (C-1) in the ^13^C NMR spectrum. In the HMBC spectrum, correlations from H-4 to C-6, 7 and from H-5 to C-3 suggested the presence of cyclopentyl moiety in **5**. These NMR spectroscopic data of **5** were quite similar to those of curcurbic acid, a hydroxylated jasmonate derivative^25^, except for the additional hydroxyl group. The position of an additional hydroxyl group was determined to be C-11, which was confirmed by the HMBC correlations from H-11 to C-8, 12 (Fig. 2). The stereochemistry was determined by the NOESY correlations between H-6, H-7 and H-2 and between H-8 and H-11 (Fig. 2). Taken together, compound **5** was defined as shown and named jasmogin A.

**Figure 3:**
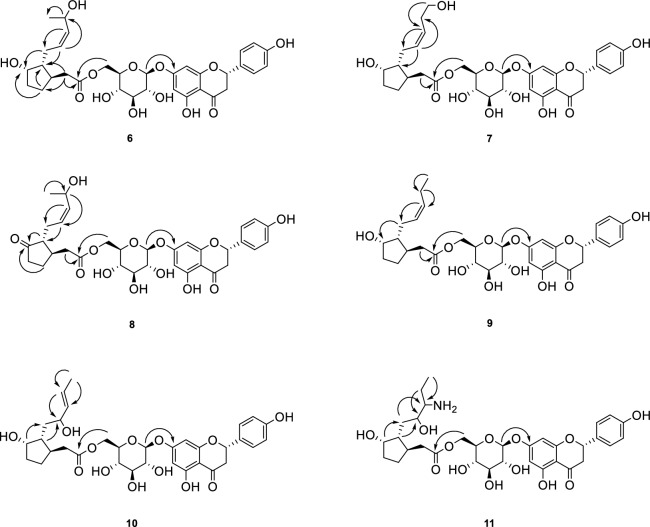
Key HMBC correlation of compounds **6–11.**

Compound **6** was purified as a white amorphous powder and assigned the molecular formula as C_33_H_40_O_13_ by its HRESI-MS (*m/z* 667.2354 [M + Na]^+^, calcd. C_33_H_40_NaO_13_ 667.2331). The NMR spectroscopic clearly showed that **6** has hydroxylated curcurbic acid moiety of **5** as a partial structure. Additionally, compound **6** was supposed to be a glycoside from the anomeric signals at [δ_H_ 4.96 (1H, d, *J* = 7.4 Hz, H-1″); δ_C_ 99.8] together with [δ_H_ 3.35–3.70 (4H, m, H-2″, 3″, 4″, 5″), 4.43 (1H, dd, *J* = 11.7, 2.1 Hz, H-6″a), 4.21 (1H, m, H-6″b); δ_C_76.4 (C-2″), 73.2 (C-3″), 70.3 (C-4″), 74.2 (C-5″), 63.2 (C-6″)] in the ^1^H and ^13^C NMR spectrum. Besides aforementioned moieties of curcurbic acid and glucose, the presence of disubstituted and tetrasubstituted aromatic rings were deduced from the signals at [δ_H_ 7.34 (2H, d, *J* = 8.0 Hz, H-2′, 6′), 6.84 (2H, d, *J* = 8.0 Hz, H-3′, 5′); δ_C_ 129.5 (C-1′), 128.9 (C-2′, 6′), 157.7 (C-4′), 115.0 (C-3′, 5′)] and from the signals at [δ_H_ 6.20 (1H, d, *J* = 2.1 Hz, H-6), 6.23 (1H, d, *J* = 2.1 Hz, H-8); δ_C_ 163.2 (C-5), 96.7 (C-6), 165.5 (C-7), 95.7 (C-8), 163.5 (C-9), 103.6 (C-10)], respectively, in the ^1^H and ^13^C NMR spectrum. From these two aromatic rings and together with additional signals at [δ_H_ 5.40 (1H, dd, *J* = 13.0, 3.0 Hz, H-2), δ_C_ 79.3 (C-2)], [δ_H_ 3.17 (1H, dd, *J* = 17.0, 13.0 Hz, H-3a), 2.77 (1H, dd, *J* = 17.0, 3.0, H-3b); δ_C_ 43.1 (C-3)] and carbonyl signal at δ_C_ 197.2 (C-4), compound **6** was supposed as a flavanone derivative, which was identified as naringenin (**1**)^23^. Taken together, **6** was suggested as a flavanone glycoside consisting of naringenin, glucose and curcurbic acid moieties. The linkage of each unit was determined by cross peaks between H-1″ of glucose and C-7 of naringenin, and between H-6″ of glucose and C-1‴ of curcurbic acid moiety in the HMBC spectrum (Fig. 3). Collectively, compound **6** was defined as shown and named jasmoflagin A.

Compound **7** was purified as a white amorphous powder and showed an HRESI-MS ion at *m/z* 667.2354 ([M + Na]^+^, calcd 667.2361) for C_33_H_40_NaO_13_. The spectroscopic data of **7** were quite similar to those of **6**, which suggested that **7** is also a curcurbinoyl derivative of naringenin glycoside. Careful comparison of ^1^H and ^13^C NMR data of **7** with those of **6** showed the differences in the chemical shifts of H-11 and H-12. The hydroxymethine and methyl signals at δ_H_ 4.62 (H-11) and 1.20 (H_3_-12) in **6** were replaced by methylene at [δ_H_ 2.37 (1H, m, H-11a) and 2.25 (1H, m, H-11b); δ_C_ 30.3] and hydroxymethylene at δ_H_ 3.56 (2H, m, H-12); δ_C_ 61.2] in **7**. The correlation between H-10‴ and C-11‴ and between H-11‴ and C-12‴ confirmed the attachment of hydroxyl group to C-12 (Fig. 3). Taken together, compound **7** was determined as shown and named jasmoflagin B.

Compound **8** was also purified as a white amorphous powder. The molecular formula was established as C_33_H_38_O_13_ from an HRESI-MS ion at m/z 665.2200 ([M + Na]^+^, calcd 665.2204). The ^1^H NMR spectrum of **8** were quite similar to those of **6**, except for the disappearance of hydroxymethine proton at δ_H_ 4.05 (H-6‴) of **6**. Additional carbonyl signal at δ_C_ 220.1 in the ^13^C NMR spectrum suggested that **8** is a jasmonate derivative of naringenin glycoside. Further HMBC correlation from H-4‴, H-5‴, H-7‴ to C-6‴ confirmed the presence of carbonyl moiety at C-6‴ (Fig. 3). Taken together, compound **8** was determined as shown and named jasmoflagin C.

Compound **9** was purified as a white amorphous powder and showed an HRESI-MS ion at m/z 651.2404 ([M + Na]^+^, calcd 651.2412) for C_33_H_40_NaO_12_. The spectroscopic data of **9** suggested that **9** is also a curcurbinoyl derivative of naringenin glycoside. However, on the contrary to **6** and **7**, hydroxymethine signals in curcurbic acid were not observed in the ^1^H and ^13^C NMR data of **9**. Further analysis demonstrated that the hydroxylmethine signals at [δ_H_ 4.62 (1H, m, H-11); δ_C_ 62.5] in **6** were replaced by methylene signals at [δ_H_ 2.06 (2H, m, H-11); δ_C_ 20.1] in **9**. In addition, HMBC correlation between H-10‴ and C-11‴ and between H-11‴ and C-12‴ confirmed the detachment of hydroxyl group at C-11 in **6** (Fig. 3). Taken together, compound **9** was determined as shown and named jasmoflagin D.

Compound **10** was purified as white amorphous powder and assigned the molecular formula as C_33_H_40_O_13_, which is same as **6**. The spectroscopic data of **10** were quite similar to those of **6** and suggested **10** is comprised of naringenin glucoside and curcurbic acid with a hydroxyl group. Differences in the ^1^H and ^13^C NMR data of **10** from those of **6** were observed as downfield shift of CH_3_-12‴ from δ_H_ 1.20 to δ_H_ 1.68 and upfield shift of H-8‴ from δ_H_ 2.04, 2.31 to δ_H_ 1.60, 1.70, suggesting the change in the positions of hydroxyl group and double bond in curcurbic acid moiety. The ^1^H-^1^H COSY correlations of H-8‴/H-9‴ and H-11‴/H-12‴ together with HMBC correlations from H-12‴ to C-11‴ determined the position of hydroxyl group at C-9‴ (Fig. 3). Taken together, compound **10** was determined as shown and named jasmoflagin E.

Compound **11** was obtained as white amorphous powder. The molecular formula of **12** was determined as C_33_H_43_NO_13_ by its HRESI-MS (*m/z* 668.2415 [M + Li]^+^, calcd. C_33_H_43_NLiO_13_ 668.2889). The ^1^H and ^13^C NMR spectrum also proposed **11** as a curcubinoyl derivative of naringenin glucoside. The ^1^H and ^13^C NMR spectrum of **11** were comparable to those of **10**, except for the disappearance of signals for double bond of curcurbic acid in **10**. Additional methine signal at [δ_H_ 3.24 (1H, m, H-10‴); δ_C_ 74.5] and methylene signals at [δ_H_ 1.40 (2H, m, H-11‴); δ_C_ 26.2] were observed in **11**. These data suggested the presence of amine group to curcubinoyl moiety of **10**, which is also confirmed by the presence of nitrogen in **11** from MS analysis. The positions of hydroxyl and amine groups were determined to be C-9‴ and C-10‴, respectively, by the HMBC correlations from H-7‴/8‴ to C-9‴ and from H-8‴/12‴ to C-10‴ (Fig. 3). Taken together, compound **11** was defined as shown and named jasmoflagin F.

### NO inhibitory activity of isolated compounds

**Figure 4:**
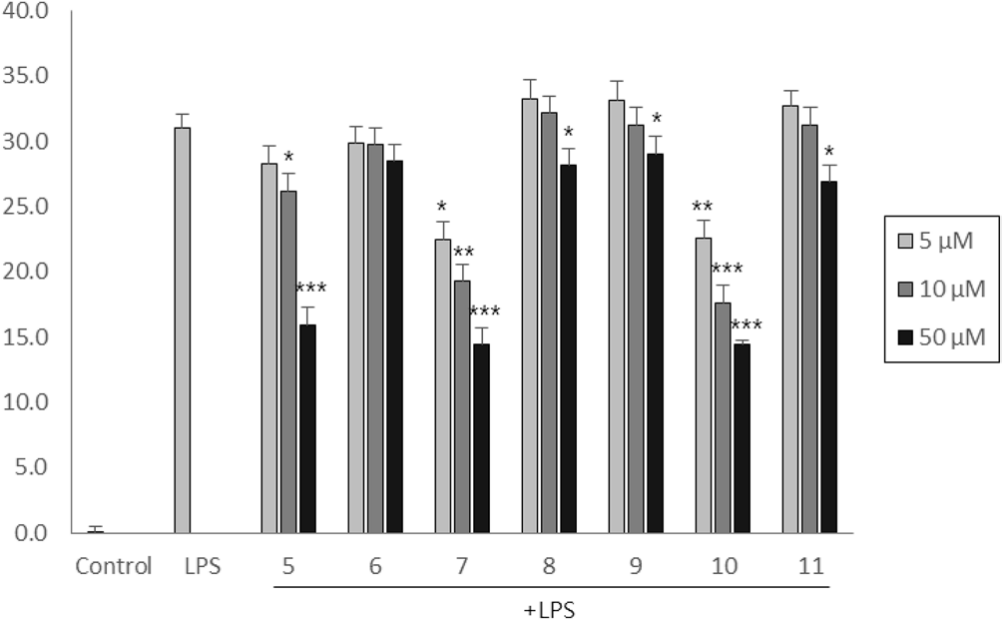
Inhibitory effects of compounds **5–11** on LPS-induced NO production in RAW 264.7 macrophage cells.

Next, we investigated the anti-inflammatory effects of newly isolated compounds by measuring the production of NO in LPS-stimulated RAW 264.7 macrophages. As shown in Fig. 4, compounds **5**, **7** and **10** dose-dependently reduced NO production stimulated by LPS without any significant cytotoxic effects at the concentration ranging from 5 to 50 μM. Compound **5,** which has only curcubinoyl moiety, inhibited NO production. However, addition of flavanone moiety to compound **5** reduced the inhibitory activity, as observed in compound **6**. Interestingly, among the curcubinoyl flavanone derivatives, compounds **7** and **10** showed stronger inhibitory activity compared to others, which suggested the importance of the position of hydroxyl group in curcurbinoyl moiety.

## Conclusion

Fractionation of using various chromatographic techniques yielded eleven compounds from the MJ-treated adventitious root cultures of wild ginseng. The chemical structures of isolated compounds were identified by spectroscopic analysis and further identified seven new compounds. The newly reported compounds are curcubinoyl derivative, named jasmogin A (**5**) and curcubinoyl-conjugated flavanone derivatives, named jasmoflagins A-F (**6**–**11**). Considering the structural similarity between methyl jasmonate and curcubinoyl moiety, addition of elicitor can affect not only the increase of biosynthesis of active metabolite, but MJ itself also participate in biosynthetic pathway as a substrate, which needs to be clarified by further study.

## Materials and methods

### General experimental procedure

IR spectra were obtained using JASCO FTIR 4100 spectrometer in CH_3_OH solvent. Optical rotations were measured on a JASCO DIP-1000 polarimeter (Tokyo, Japan). HRESIMS data were measured on maXis 4G (Bruker) and LCQ Fleet (Thermoscientific), respectively. NMR spectra were recorded on a Bruker Avance 400, 500 and 800 MHz spectrometers using CD_3_OD as solvent. Silica gel (200–400 mesh, Merck), Sephadex LH-20 (20–100 μm, Sigma) and Diaion HP-20P (Mitsubishi Kasei, Japan) for column chromatography. TLC was performed on silica gel 60 F_254_ (0.2 mm, Merck) or silica gel 60 RP-18 F_254S_ (0.2 mm, Merck), and spots were detected by a 10% vanillin-H_2_SO_4_ in EtOH spray reagent. MPLC was performed on a Biotage Isolera Prime chromatography system and a Lichroprep RP-18 column (40–63 μm). Semi-prep HPLC was performed using a Waters system (three 515 pumps and a 996 photodiode array detector) with a Phenomenex Gemini-NX 5 μ C18 110A column (USA). Details of NMR and HPLC methodology as well as NMR/NOESY spectra of new compounds are present in Supplementary Information.

### Plant material

Wild ginseng was collected at Mt. Ohdae of Korea by the government certificated digger. It was identified by the Emeritus Prof. Kee-Yoeup Paek and certificated by Korea ginseng institutes. The permissions were obtained from concerned authorities for collection and use of sample. And all methods were performed in accordance with the relevant regulations.

Adventitious root cultures of wild ginseng (*P. ginseng*) were produced from a 100-year-old wild ginseng through callus culture as we described previously^20^. The root cultures were proliferated in a 5 L airlift balloon type bioreactor containing 4.0 L Murashige and Skoog (MS) liquid medium (3/4 strength) supplemented with 5.0 mg/L IBA, 0.1 mg/L kinetin, and 5% (w/v) sucrose for seven weeks. The stock solution of MJ was prepared in ethanol as 50 mM and MJ were added to the culture as an elicitor as final concentration of 100 μM, one week before harvest. The adventitious roots were harvested from the culture and washed three times with distilled water to remove the medium on the surface of the adventitious roots. Then, it was immediately frozen with liquid nitrogen and stored in deep-freezer at − 70 °C, and then freeze-dried before further experiments. A voucher specimen (CBNU-WGAR2014) was deposited at the Herbarium of the College of Pharmacy, Chungbuk National University, Korea.

### Extraction and isolation of compounds

The dried MJ-treated wild ginseng adventitious root cultures (5.0 kg) were extracted twice with 25 L of 80% MeOH for 24 h at room temperature. The methanol extract (1.7 kg) was suspended in H_2_O and partitioned successively with *n*-hexane, CH_2_Cl_2_, EtOAc and *n*-BuOH to yield corresponding fractions.

The EtOAc fraction (WGE, 21.9 g) was subjected to MPLC over silica gel (CH_2_Cl_2_-MeCN, 1:0 → 0:1) to afford sixteen subfractions (WGE1-WGE16). WGE5 was subjected to MPLC over reverse phase silica gel (MeOH-H_2_O, 10:90 → 1:0) to give five subfractions (WGE5A- WGE5E). Compounds **9** (17.1 mg) and **10** (12.6 mg) were purified from WGE5E by semi-preparative HPLC eluting with MeCN- H_2_O (30:70). WGE9 was subjected to MPLC over reverse phase silica gel (MeOH-H_2_O, 10:90 → 1:0) to give three subfractions (WGE9A- WGE9C). Compound **3** (29.7 mg) were purified from WGE9B by semi-preparative HPLC eluting with MeCN- H_2_O (30:70). WGE15 was subjected to MPLC over reverse phase silica gel (MeOH-H_2_O, 10:90 → 1:0) to give two subfractions (WGE15A- WGE15B). Compounds **1** (19.9 mg) and **5** (13.4 mg) were purified from WGE15A by semi-preparative HPLC eluting with MeCN- H_2_O (30:70).

The *n*-BuOH fraction (WGB, 218.3 g) was subjected to HP-20 (MeOH-H_2_O, 0:1 → 1:0) to afford five subfractions (WGB1-WGB5). WGB5 was subjected to MPLC over reverse phase silica gel (CH_2_Cl_2_-MeCN, 1:0 → 0:1) to give nine subfractions (WGE5A- WGE5I). WGB5C was subjected to MPLC over silica gel (MeOH-H_2_O, 10:90 → 1:0) to give three subfractions (WGE5C1- WGE5C3). Compounds **8** (18.1 mg) and **11** (11.7 mg) were purified from WGE5C1 by semi-preparative HPLC eluting with MeCN- H_2_O (30:70). Compounds **2** (15.0 mg), **4** (15.3 mg), **6** (16.2 mg) and **7** (16.7 mg) were purified from WGE5C2 by semi-preparative HPLC eluting with MeCN- H_2_O (40:60).

***Jasmogin*** (**5**) Light yellow gum;${[\alpha ]}_{D}^{25}$ -53.9° (*c* 0.01, MeOH); IR (KBr) n_max_ 3367, 1716 cm^-1^; UV (MeOH) λ_max_ 224, 281 nm; ESIMS *m/z* 249 [M + H]^+^; HRESIMS *m/z* 251.1254 [M + Na]^+^ (calcd for C_12_H_20_NaO_4_ 251.1253); ^1^H-NMR (500 MHz, CD_3_OD) and ^13^C-NMR (225 MHz, CD_3_OD), see Table 1.

**Table 1:** NMR spectroscopic data for compound **5** (CD_3_OD).

	δ_H_	δ_C_
1		175.9
2	2.55 (dd, 14.4, 3.6), 2.15 (m)	39.1
3	2.16 (m)	38.5
4	1.88 (m), 1.64 (m)	32.8
5	2.11 (m), 1.33 (m)	28.8
6	4.15 (m)	73.4
7	1.46 (m)	50.9
8	2.29 (m), 2.24 (m)	25.5
9	5.52 (m)	128.8
10	5.42 (dd, 10.4, 8.8)	134.0
11	4.68 (dq, 8.8, 6.4)	63.2
12	1.23 (d, 6.4)	22.5

***Jasmoflagin**** A* (**6**) Light yellow gum; ${[\alpha ]}_{D}^{25}$-79.2 (*c* 0.05, MeOH); IR (KBr) n_max_ 3537, 2915, 2337, 1725, 1052 cm^-1^; UV (MeOH) λ_max_ 283, 324 nm; ESIMS *m/z* 667 [M + Na]^+^; HRESIMS *m/z* 667.2354 [M + Na]^+^ (calcd for C_33_H_40_NaO_13_ 667.2331); ^1^H-NMR (500 MHz, CD_3_OD) and ^13^C-NMR (175 MHz, CD_3_OD), see Tables 2 and 3.

**Table 2:** ^1^H NMR spectroscopic data for compounds **6–11** (CD_3_OD).

No	**6**	**7**	**8**	**9**	**10**	**11**
2	5.40, dd (13.0, 3.0)	5.41, dd (12.5, 3.5)	5.41, dd (12.6, 3.5)	5.38, dd (13.0, 2.8)	5.42, dd (13.0, 3.0)	5.42, dd (12.7, 2.9)
3	3.17, dd (17.0, 13.0)	3.17, dd (17.0, 12.5)	3.18, dd (17.5, 12.6)	3.14, dd (17.1, 13.0)	3.16, dd (17.0, 13.0)	3.18, dd (17.1, 12.7)
	2.77, dd (17.0, 3.0)	2.77, dd (17.0, 3.5)	2.76, dd (17.5, 3.5)	2.75, dd (17.1, 2.8)	2.76, dd (17.0, 3.0)	2.78, dd (17.1, 2.9)
6	6.20, d (2.1)	6.20, d (2.2)	6.20, d (2.2)	6.20, d (2.3)	6.21, d (2.1)	6.21, d (2.1)
8	6.23, d (2.1)	6.23, d (2.2)	6.25 d (2.2)	6.23, d (2.3)	6.24, d (2.1)	6.24, d (2.1)
2′/6′	7.34, d (8.0)	7.34, d (8.5)	7.34, d (8.5)	7.33, d (8.5)	7.35, d (8.3)	7.35, d (8.6)
3′/5′	6.84, d (8.0)	6.84, d (8.5)	6.83, d (8.5)	6.84, d (8.5)	6.85, d (8.3)	6.85, d (8.6)
1″	4.96, d (7.4)	4.96, d (7.4)	4.97, d (7.5)	4.95, d (7.5)	4.97, d (7.4)	4.96, d (7.5)
2″	3.48 m	3.48 m	3.50 m	3.47 m	3.49 m	3.48 m
3″	3.46 m	3.47 m	3.47 m	3.46 m	3.47 m	3.46 m
4″	3.34 m	3.34 m	3.33 m	3.34 m	3.34 m	3.33 m
5″	3.70 m	3.71 m	3.71 m	3.69 m	3.72, t (9.8)	3.72 m
6″	4.43, dd (11.7. 2.1)	4.43, dd (11.9, 2.1)	4.51, dd (11.8, 2.1)	4.42, dd (11.8, 1.9)	4.48 dd (11.8, 2.1)	4.47 dd (11.8, 1.9)
	4.21 m	4.22 m	4.18 m	4.21 m	4.15 m	4.15 m
2‴	2.52 m	2.54 m	2.67 m	2.54 m	2.33, m	2.35 m
	2.22 m	2.20 m	2.36 m	2.18 m	2.31, m	2.30 m
3‴	2.08 m	2.08 m	2.19 m	2.06 m	1.96 m	1.96 m
4‴	1.99 m	1.74 m	1.38 m	1.96 m	1.81 m	1.85 m
	1.19 m	1.53 m	2.10 m	1.20 m	0.99 m	1.52 m
5‴	1.75 m	1.98 m	2.30 m	1.73 m	1.87 m	1.83 m
	1.54 m	1.18 m	2.31 m	1.55 m	1.47 m	1.12 m
6‴	4.05 m	4.07 m	–	4.07 m	4.48 m	4.48 m
7‴	1.24 m	1.24 m	1.71 m	1.23 m	2.14 m	2.13 m
8‴	2.31 m	2.20 m	2.03 m	2.12 m	1.70 m	1.70 m
	2.04 m	2.09 m	2.23 m	2.12 m	1.60 m	1.62 m
9‴	5.43 m	5.51 m	5.26 m	5.37 m	4.15 m	3.69 m
10‴	5.41 m	5.41 m	5.40 m	5.36 m	5.37 m	3.24 m
11‴	4.62 m	2.37 m, 2.25 m	4.58 m	2.06 m	5.65 m	1.40 m
12‴	1.20, d (6.4)	3.56 m	1.18, d (6.3)	0.96, t (7.5)	1.68, d (6.3)	0.97, dd (14.1, 7.1)

**Table 3:** ^13^C NMR spectroscopic data for compounds **6–11** (CD_3_OD).

	**6**	**7**	**8**	**9**	**10**	**11**
2	79.3	79.3	79.4	79.3	79.4	79.4
3	43.1	43.1	43.1	43.0	43.1	43.1
4	197.1	197.1	197.2	197.1	197.2	197.2
5	163.2	163.2	163.2	163.2	163.3	163.3
6	96.7	96.7	96.8	96.7	96.7	96.7
7	165.5	165.5	165.4	165.5	165.5	165.4
8	95.7	95.7	95.7	95.7	95.6	95.6
9	163.5	163.5	163.4	163.5	163.5	163.5
10	103.6	103.6	103.6	103.6	103.6	103.6
1′	129.5	129.5	129.5	129.5	129.5	129.6
2′/6′	127.6	127.5	127.6	127.6	127.6	127.8
3′/5′	115.0	115.0	115.1	115.0	115.0	115.0
4′	157.7	157.7	157.8	157.7	157.8	157.7
1″	99.8	99.8	99.8	99.8	99.8	99.9
2″	76.4	76.4	76.3	76.4	76.3	76.3
3″	73.2	73.2	73.2	73.2	73.2	73.2
4″	70.3	70.3	70.3	70.3	70.4	70.4
5″	74.2	74.2	74.2	74.2	74.2	74.2
6″	63.2	63.2	63.5	63.2	63.5	63.5
1‴	173.4	173.5	172.3	173.5	173	173.0
2‴	38.4	38.7	38.2	43.0	38.9	39.0
3‴	38.2	38.4	37.7	38.4	41.9	42.0
4‴	28.8	32.5	26.6	28.7	31.5	32.2
5‴	32.3	28.7	25.0	32.7	32.4	31.4
6‴	73.2	73.4	220.1	73.6	84.5	84.8
7‴	50.7	50.7	53.5	50.8	49.5	49.5
8‴	25.7	25.4	37.0	25.2	38.2	33.7
9‴	130.1	130.4	126.2	127.8	79.1	81.5
10‴	133.8	126.0	135.6	131.6	130.8	74.5
11‴	62.5	30.3	62.8	20.1	127.7	26.2
12‴	22.2	61.2	22.4	13.3	16.5	9.2

***Jasmoflagin B*** (**7**) Light yellow gum; ${[\alpha ]}_{D}^{25}$-97.1 (*c* 0.05, MeOH); IR (KBr) n_max_ 3698, 3250, 2044, 1662, 1362, 802 cm^-1^; UV (MeOH) λ_max_ 212, 281, 327 nm; ESIMS *m/z* 667 [M + Na]^+^; HRESIMS *m/z* 667.2354 [M + Na]^+^ (calcd for C_33_H_40_NaO_13_ 667.2361); ^1^H-NMR (700 MHz, CD_3_OD) and ^13^C-NMR (175 MHz, CD_3_OD), see Tables 2 and 3.

***Jasmoflagin C*** (**8**) Light yellow gum; ${[\alpha ]}_{D}^{25}$ -34.2 (*c* 0.01, MeOH); IR (KBr) n_max_ 3861, 3563, 2360, 1515, 1176 cm^-1^; UV (MeOH) λ_max_ 215, 283, 327 nm; ESIMS *m/z* 667 [M + Na]^+^; HRESIMS *m/z* 667.2360 [M + Na]^+^ (calcd for C_33_H_40_NaO_13_ 667.2361); ^1^H-NMR (700 MHz, CD_3_OD) and ^13^C-NMR (175 MHz, CD_3_OD), see Tables 2 and 3.

***Jasmoflagin D*** (**9**) Light yellow gum; ${[\alpha ]}_{D}^{25}$-38.5 (*c* 0.05, MeOH); IR (KBr) n_max_ 3876, 3617, 2869, 1639, 887 cm^-1^; UV (MeOH) λ_max_ 212, 281, 389 nm; ESIMS *m/z* 667 [M + Na]^+^; HRESIMS *m/z* 651.2404 [M + Na]^+^ (calcd for C_33_H_40_NaO_12_ 651.2412); ^1^H-NMR (50 MHz, CD_3_OD) and ^13^C-NMR (100 MHz, CD_3_OD), see Tables 2 and 3.

***Jasmoflagin E*** (**10**) Light yellow gum; ${[\alpha ]}_{D}^{25}$-92.2 (*c* 0.05, MeOH); IR (KBr) n_max_ 3751, 3311, 2331, 1662, 824 cm^-1^; UV (MeOH) λ_max_ 283, 325 nm; ESIMS *m/z* 667 [M + Na]^+^; HRESIMS *m/z* 651.2334 [M + Li]^+^ (calcd for C_33_H_40_LiO_13_ 651.2624); ^1^H-NMR (400 MHz, CD_3_OD) and ^13^C-NMR (100 MHz, CD_3_OD), see Tables 2 and 3.

***Jasmoflagin F*** (**11**) Light yellow gum; ${[\alpha ]}_{D}^{25}$-64.8 (*c* 0.05, MeOH); IR (KBr) n_max_ 3706, 3494, 2969, 1577, 1058, 833 cm^-1^; UV (MeOH) λ_max_ 211, 283, 325 nm; ESIMS *m/z* 662 [M + H]^+^; HRESIMS *m/z* 668.2415 [M + Li]^+^ (calcd for C_33_H_43_NLiO_13_ 668.2889); ^1^H-NMR (700 MHz, CD_3_OD) and ^13^C-NMR (175 MHz, CD_3_OD), see Tables 2 and 3.

### Measurement of LPS-induced NO production

Inhibitory effect of compounds on lipopolysaccharide (LPS)-induced nitric oxide (NO) production was assessed using RAW264.7 macrophage cell lines. RAW 264.7 cells were treated with 1 μg/ml LPS in the presence or absence of compounds. After 24 h incubation, the cell medium was mixed with Griess reagent and the amount of NO formed was determined by measuring the absorbance at 550 nm in an ELISA reader. Cell viability of the remaining cells was determined by MTT assay.

## Supplementary Information

Supplementary Information.
