# Supplementary material for: Curcubinoyl flavonoids from wild ginseng adventitious root cultures
Source: Sci Rep. 2021 Jun 9;11:12212. doi: 10.1038/s41598-021-91850-8 (PMC8190163; doi:10.1038/s41598-021-91850-8)

**Curcubinoyl flavonoids from wild ginseng adventitious root cultures**

Qing Liu^1^, Seon Beom Kim^1^, Yang Hee Jo^1^, Jong Hoon Ahn^1^, Ayman Turk^1^, Da Eun Kim^2^, Bo Yoon Chang^2^, Sung Yeon Kim^2^, Cheol-Seung Jeong^3^, Bang Yeon Hwang^1^, So-Young Park^3,*^ and Mi Kyeong Lee^1,*^

*^1^College of Pharmacy, Chungbuk National University, Cheongju 28160, Republic of Korea*

*^2^ College of Pharmacy, Wonkwang University, Iksan 54538, Republic of Korea*

*^3^Department of Horticultural Science, Chungbuk National University, Cheongju 28644, Republic of Korea*

**[Experimental]**

**Condition of NMR analysis**

NMR was measured as follows with little modification.

EXPNO 201602299

PROCNO 1

Time 10.34

INSTRUM spect

PROBHD 5 mm DUL 13C-1

PULPROG zg30

TD 65536

NS 16

DS 2

SWH 10330.578 Hz

FIDRES 0.157632 Hz

AQ 3.1720407 sec

RG 128

DW 48.400 usec

DE 6.50 usec

TE 298.0 K

D1 1.00000000 sec

TD0 1

**Condition of HPLC analysis**

For the preparation of compounds, Semi-prep HPLC was performed using a Waters system (three 515 pumps and a 996 photodiode array detector) with a Phenomenex Gemini-NX 5 μ C18 110A column (USA). HPLC was run at room temperature using the mobile phase as indicated with a flow rate of 2.0 ml/min.


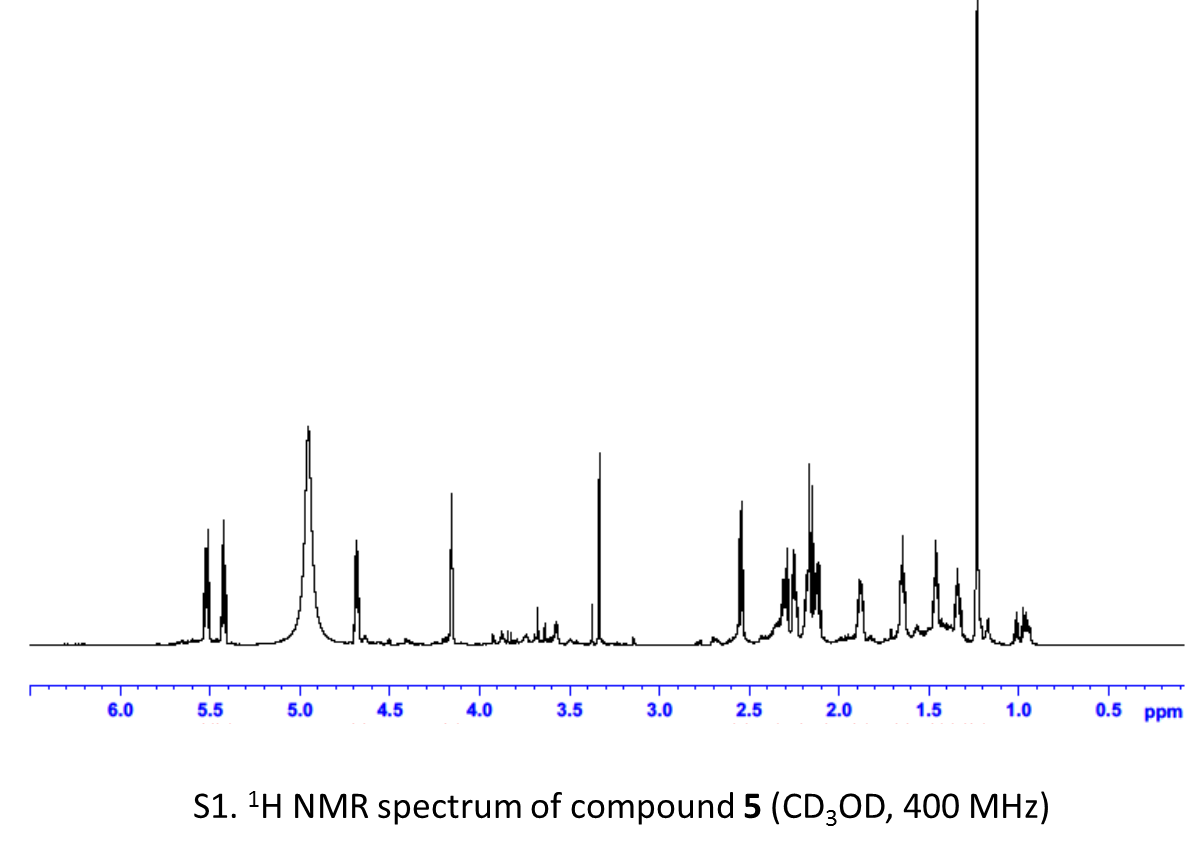


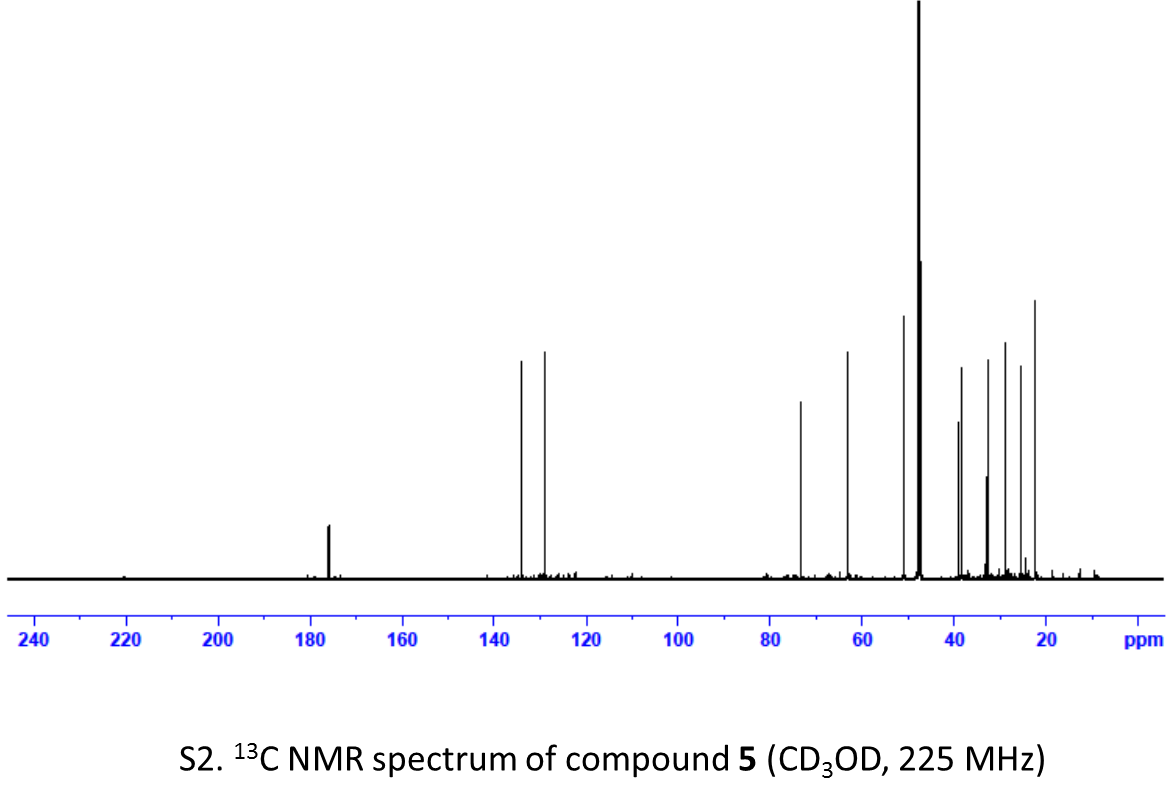


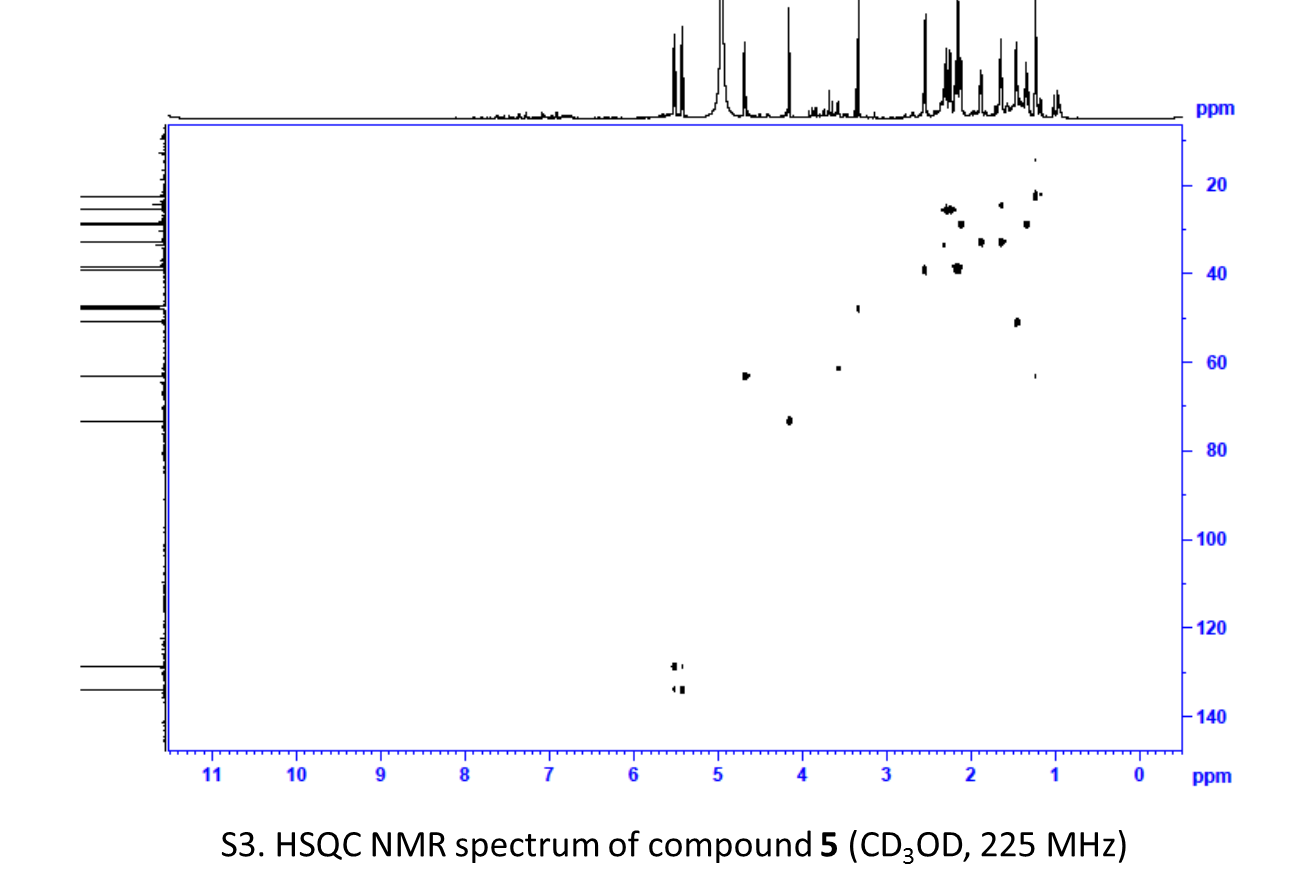


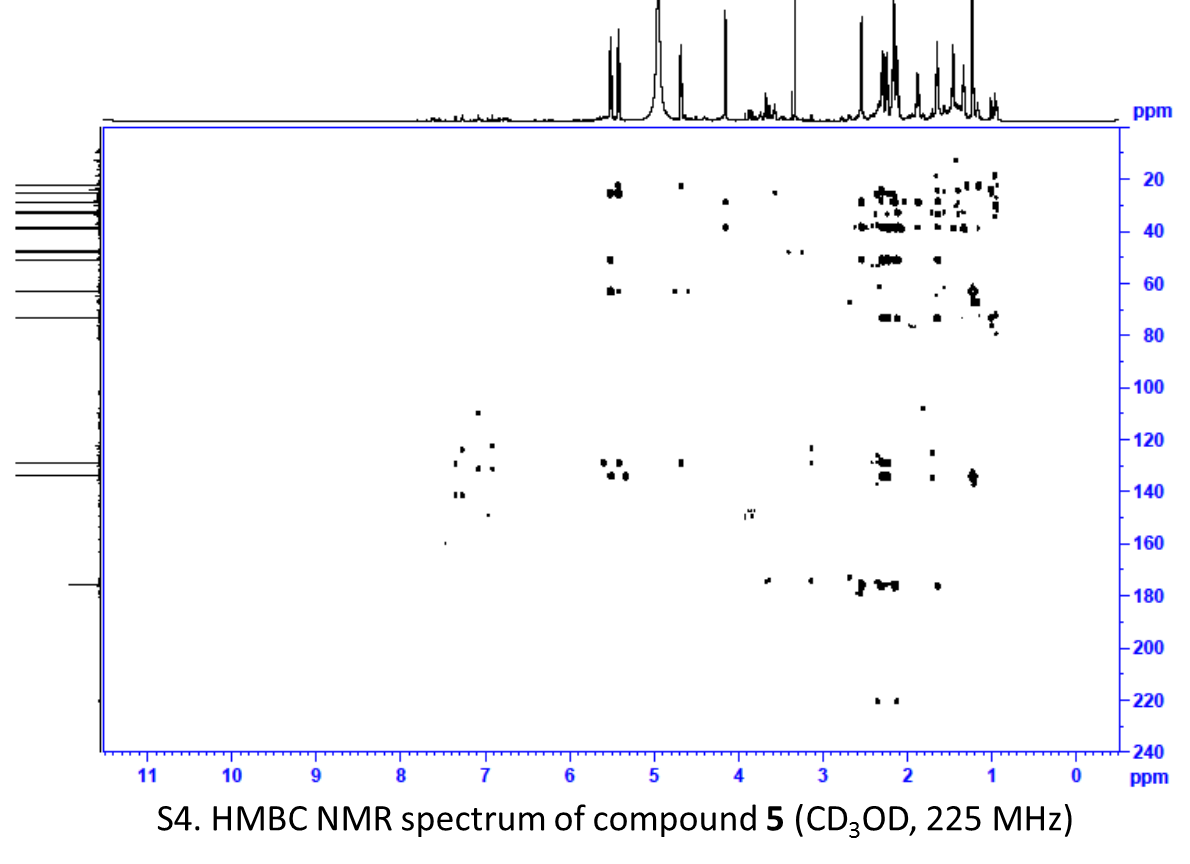


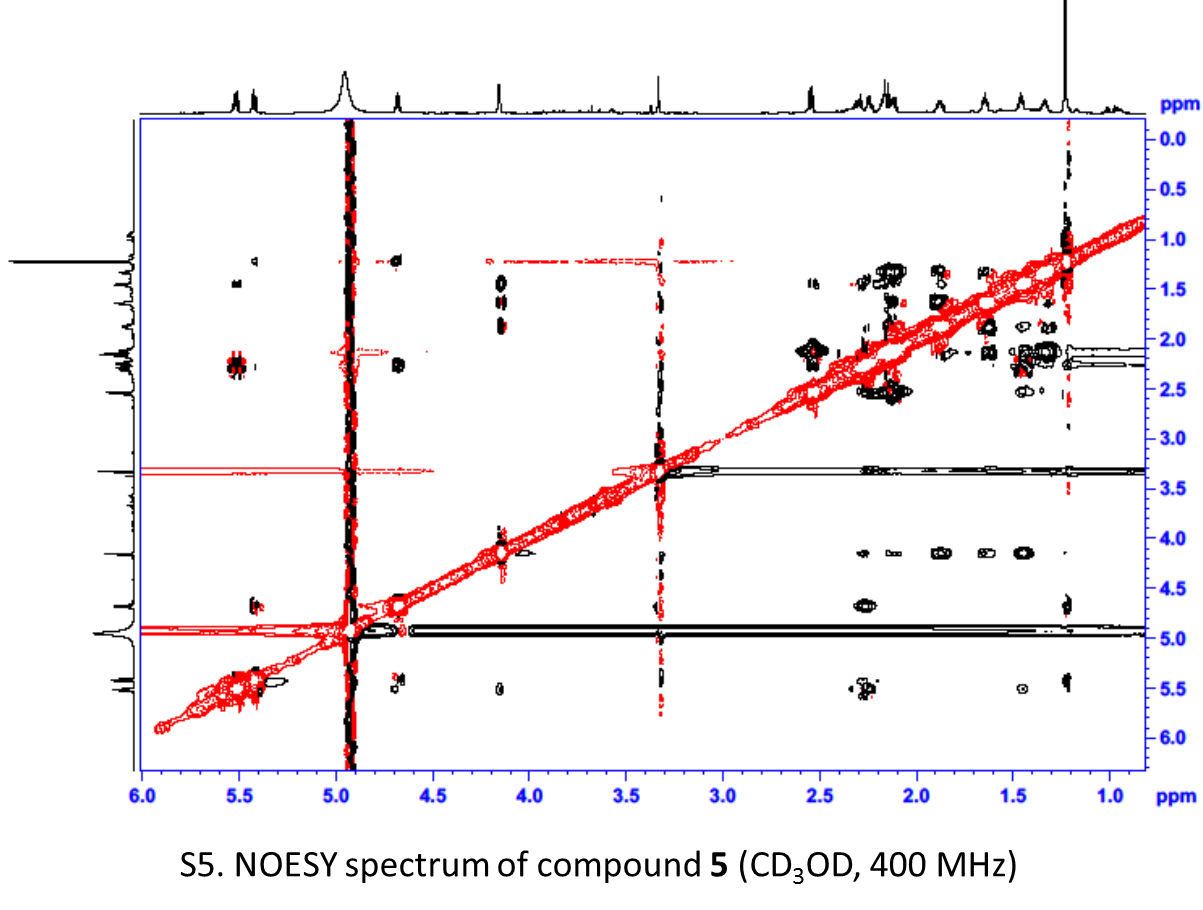


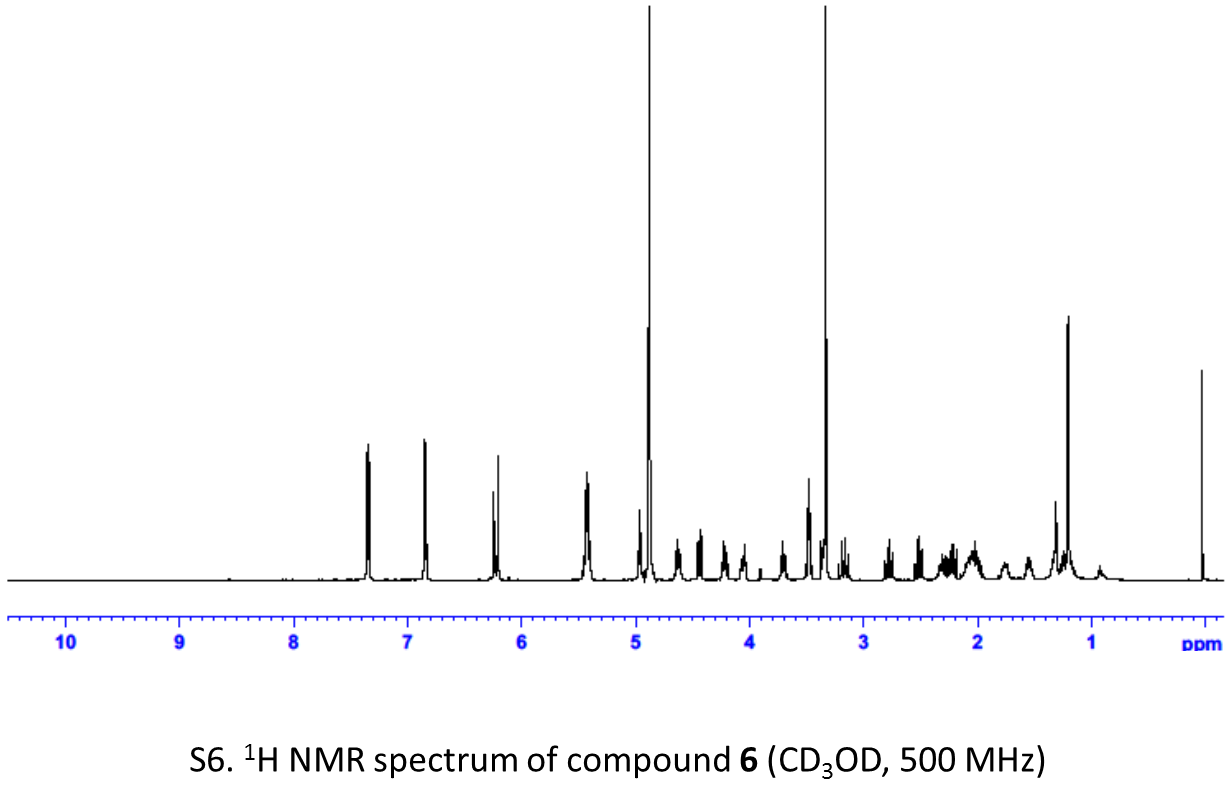


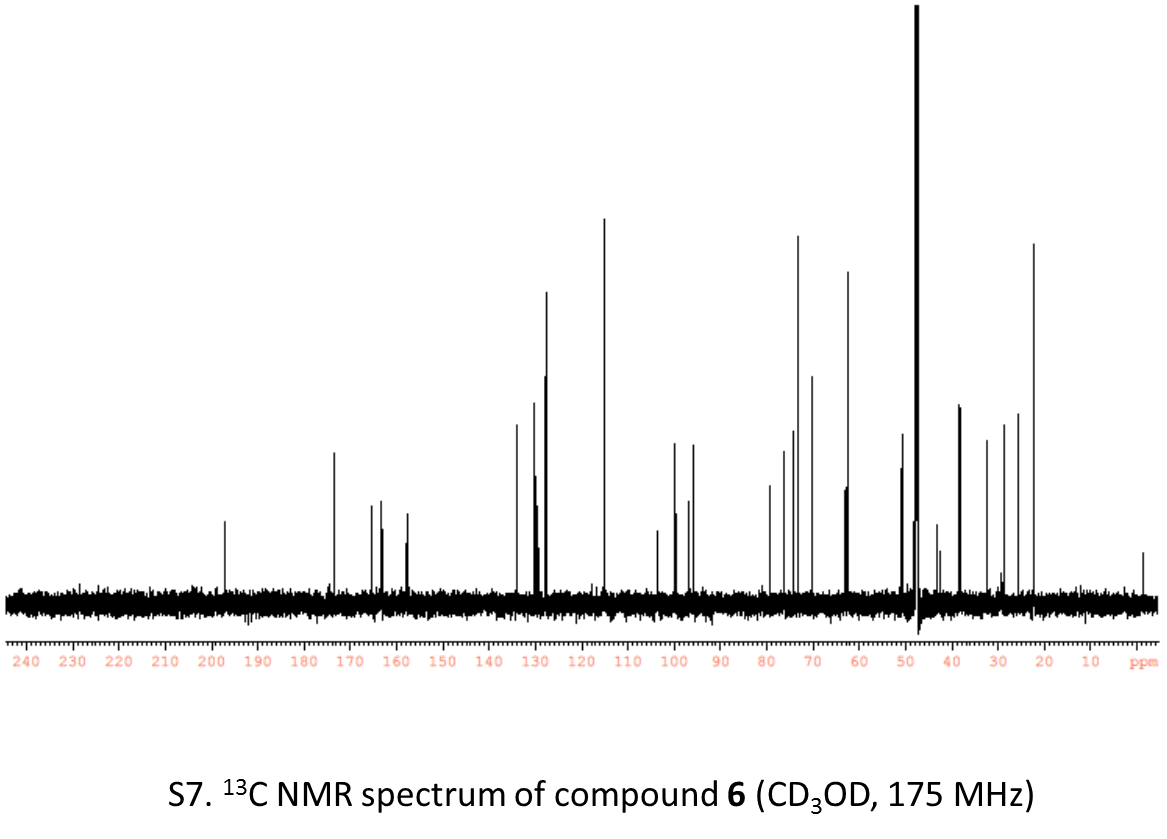


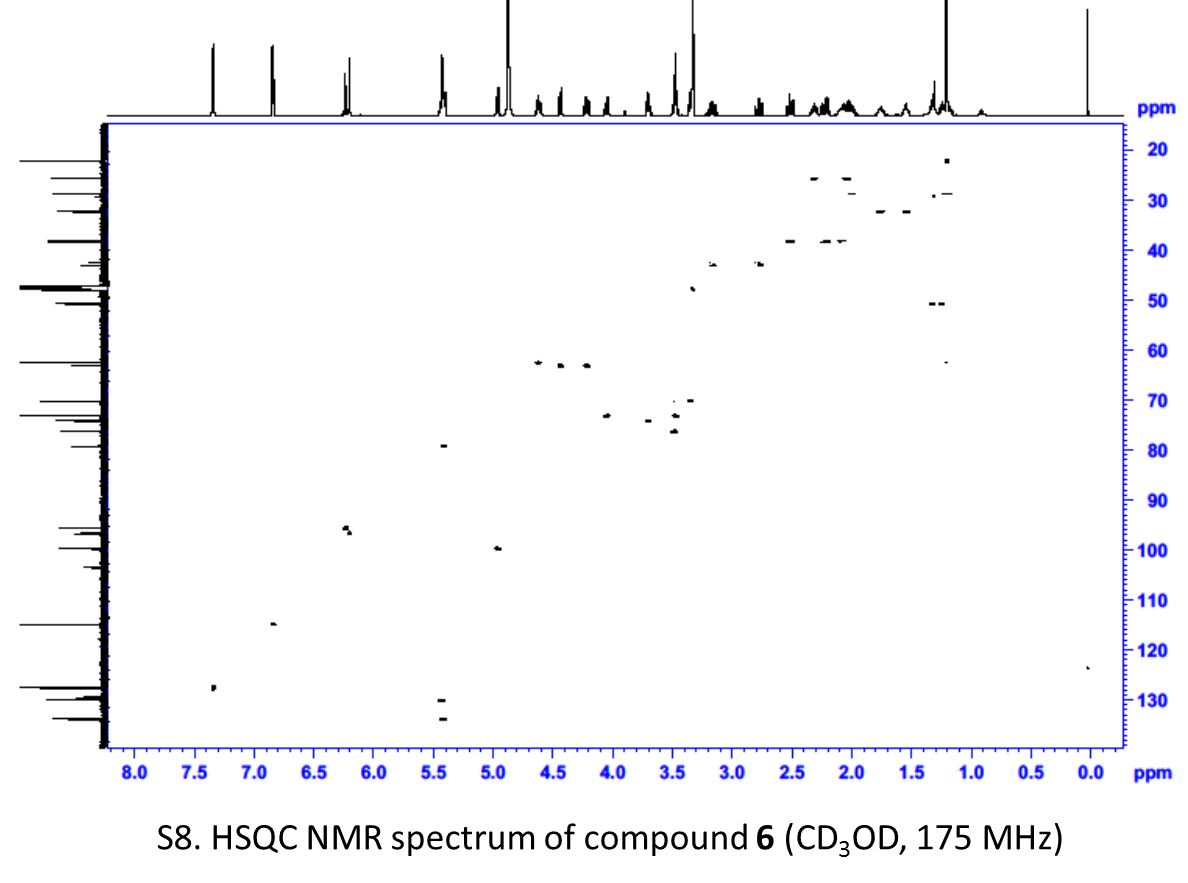


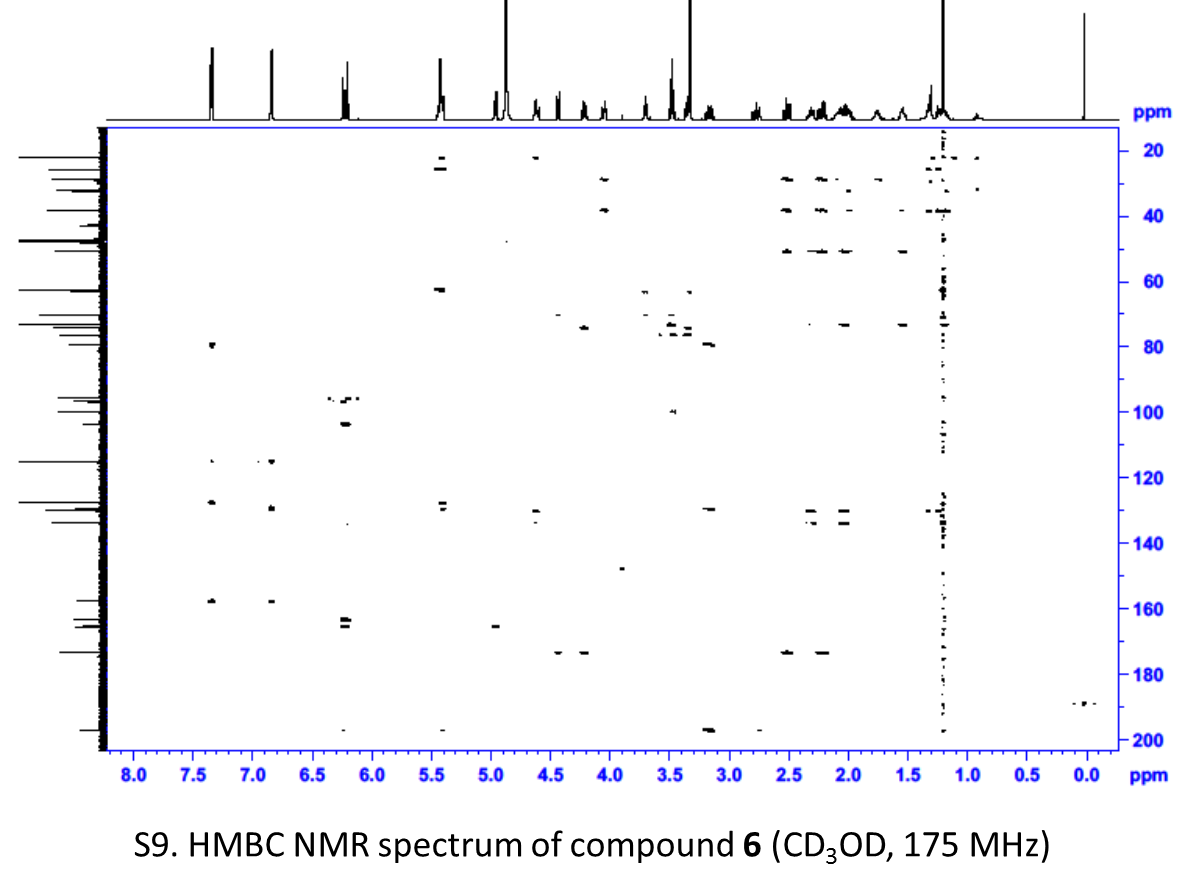

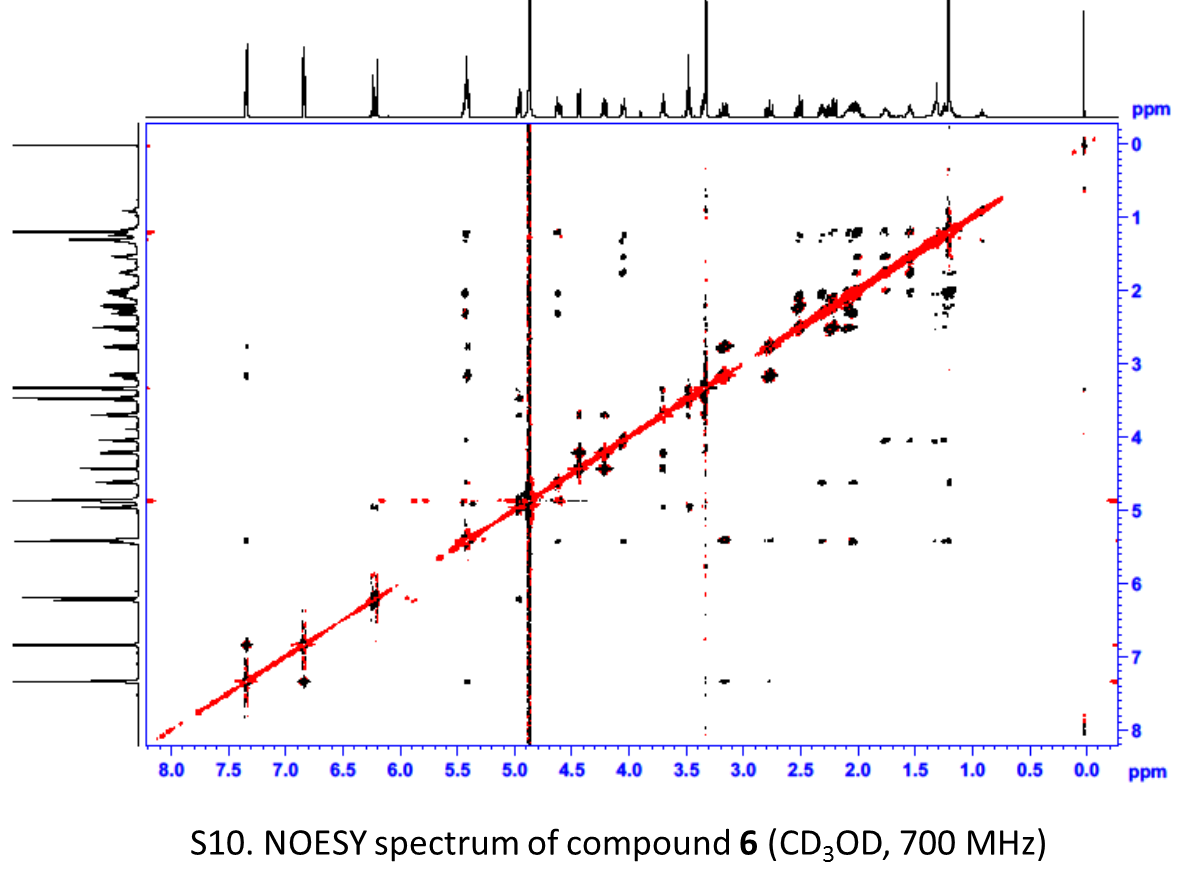


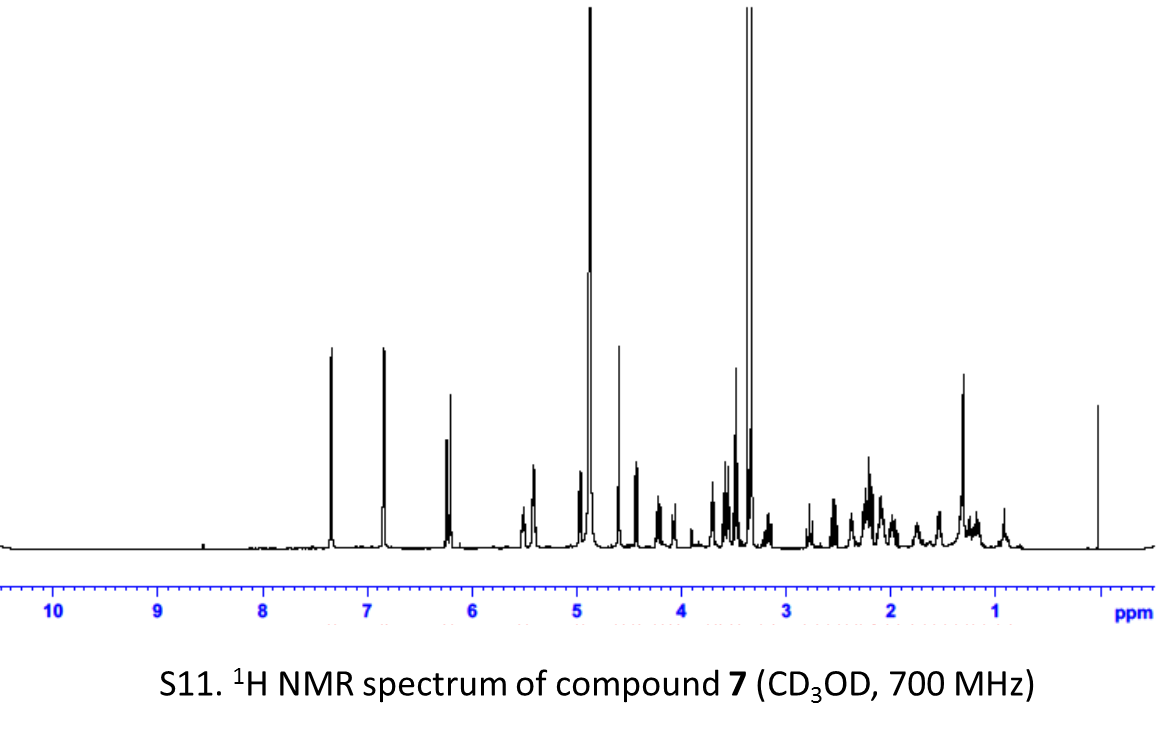


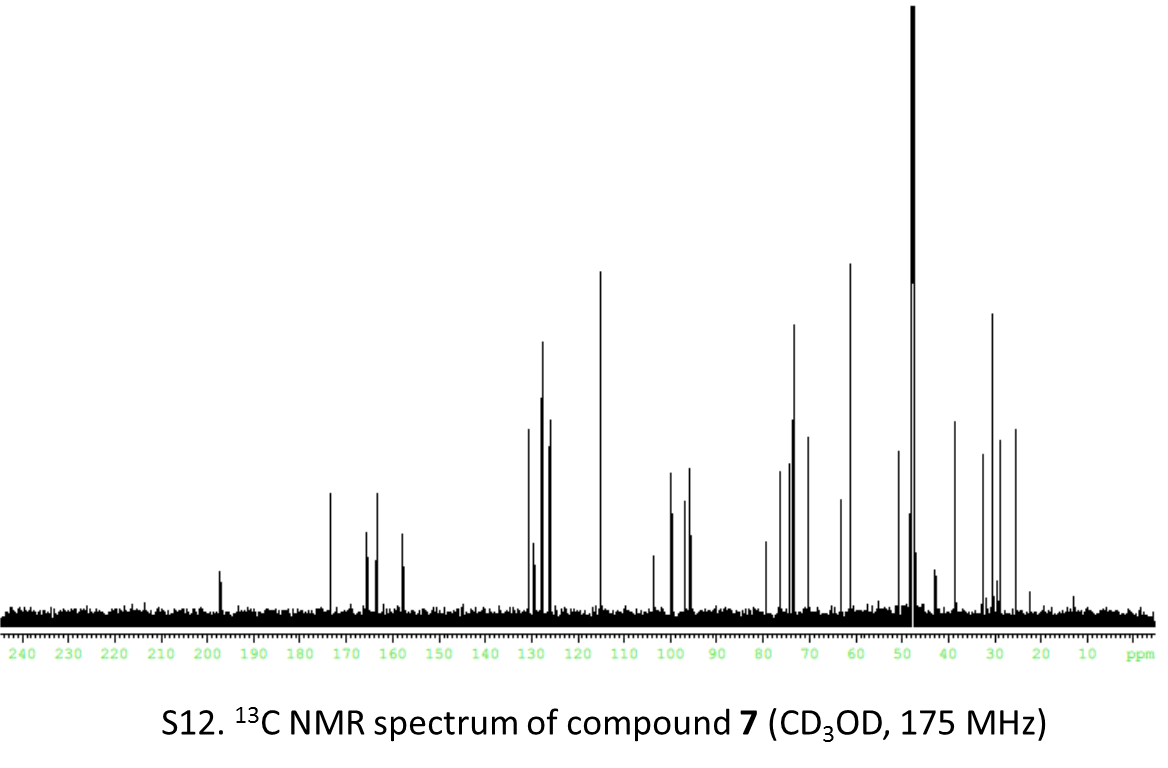


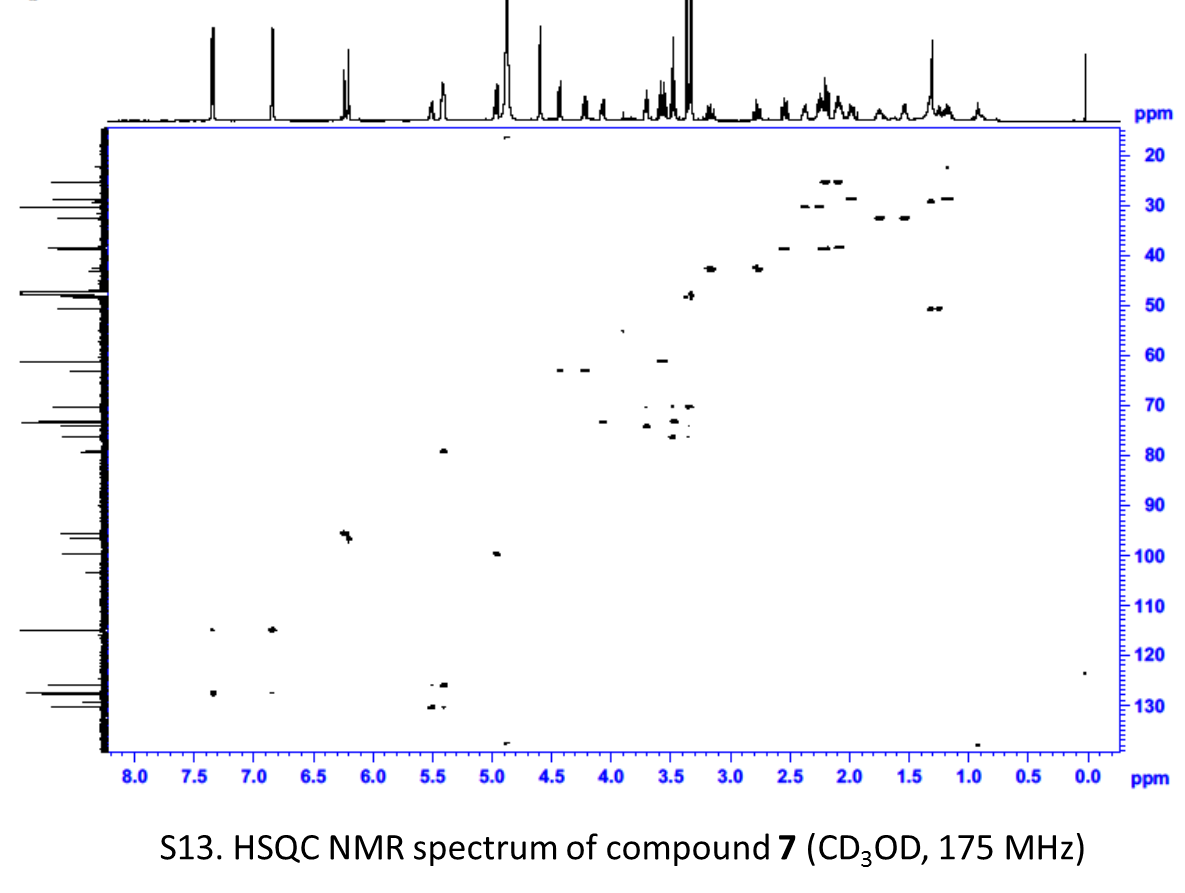

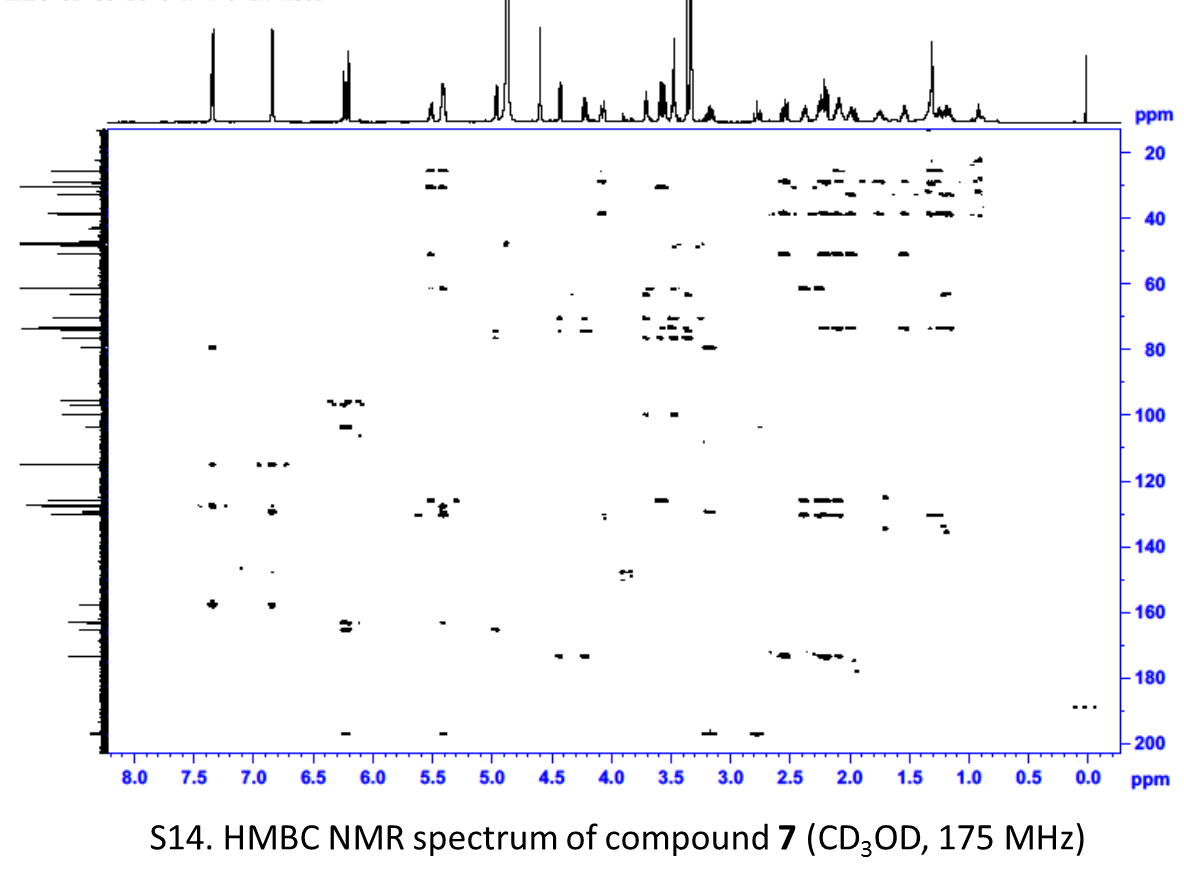


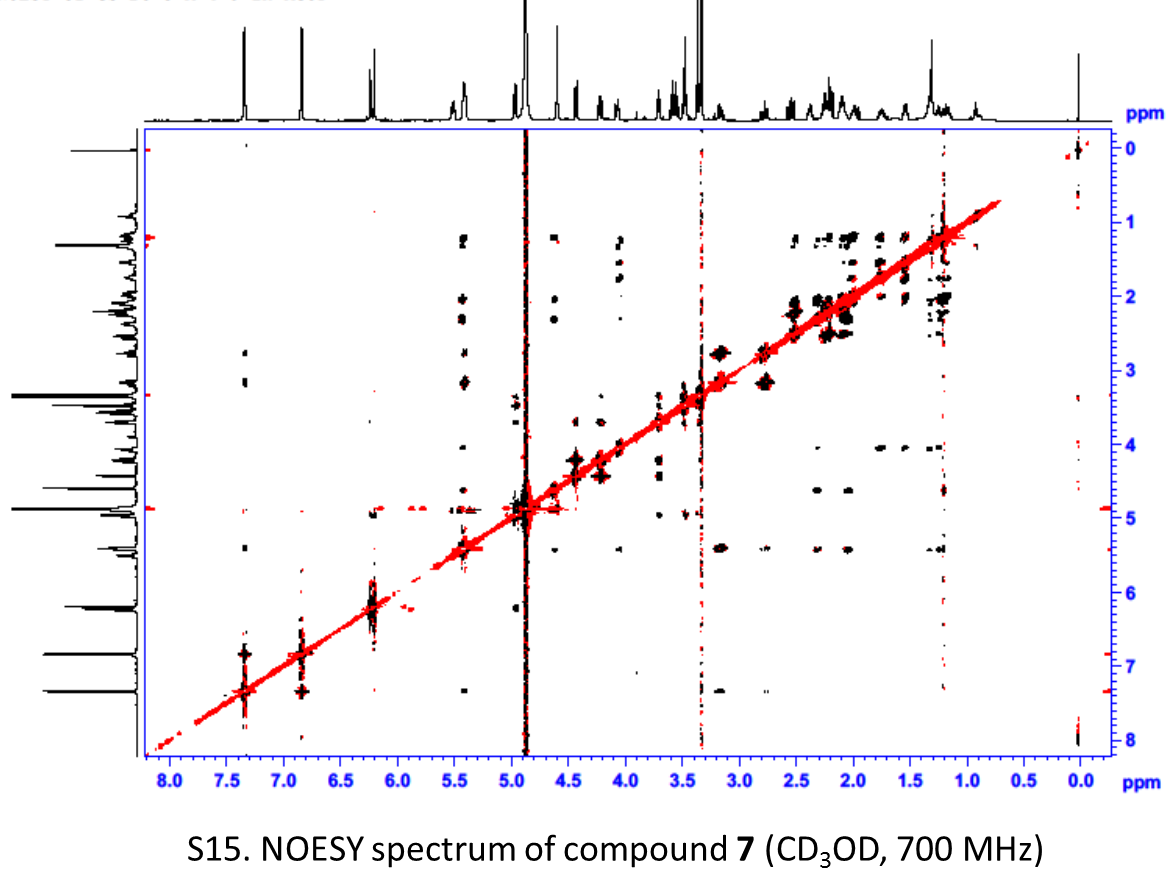


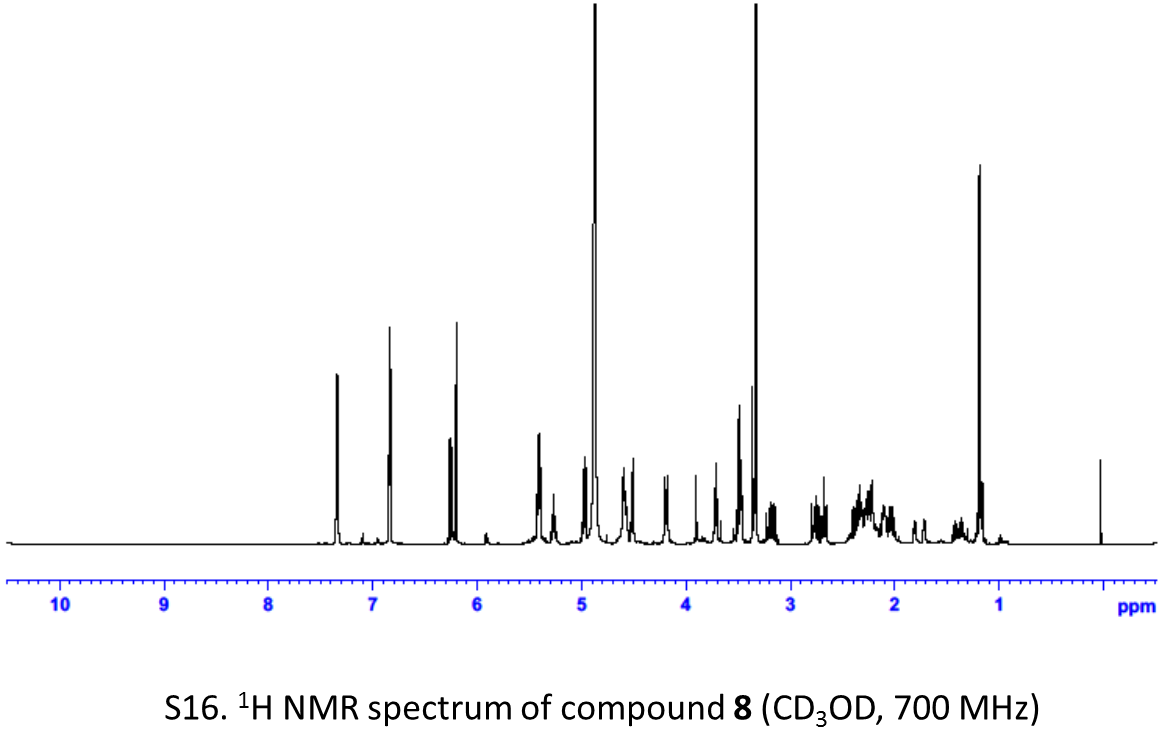


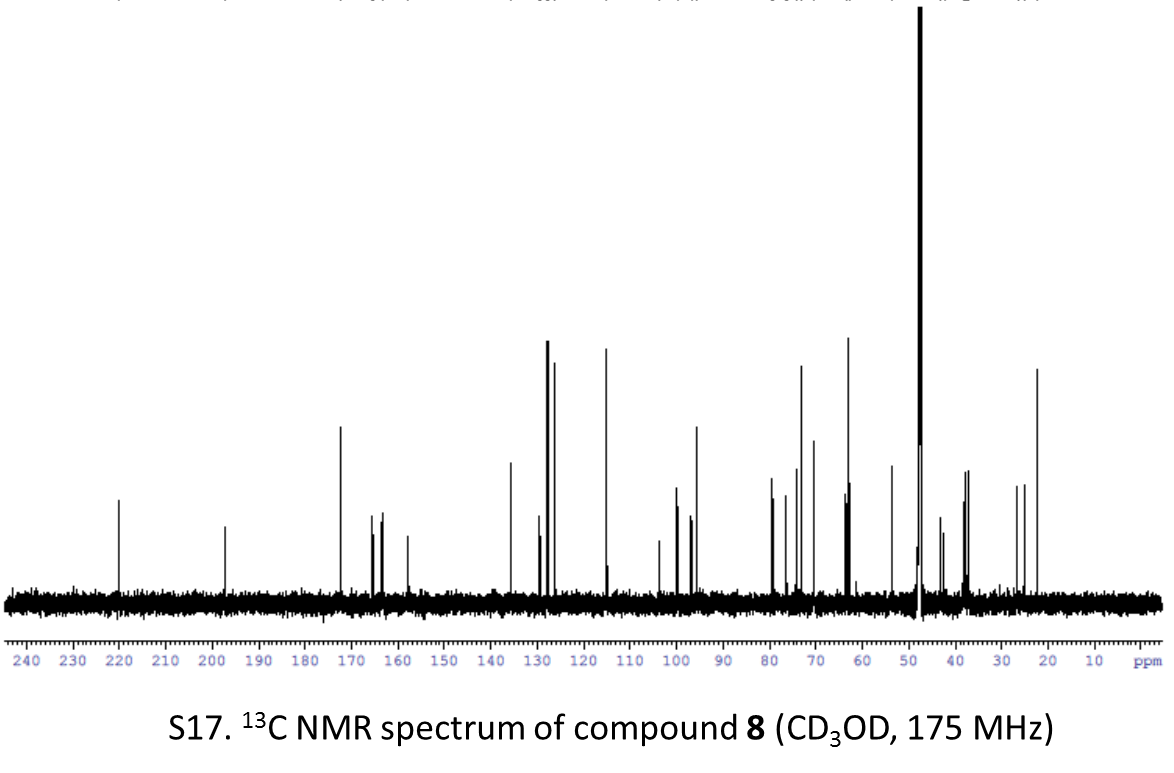


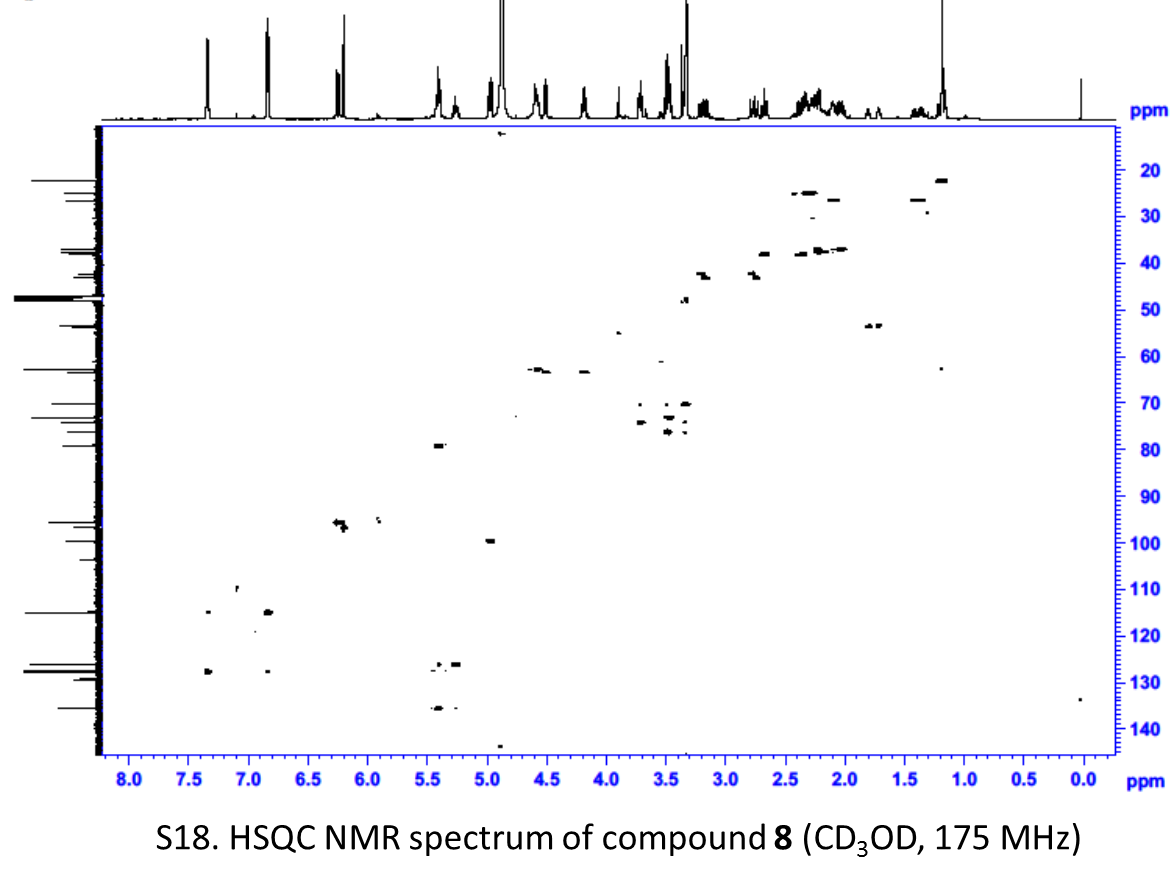


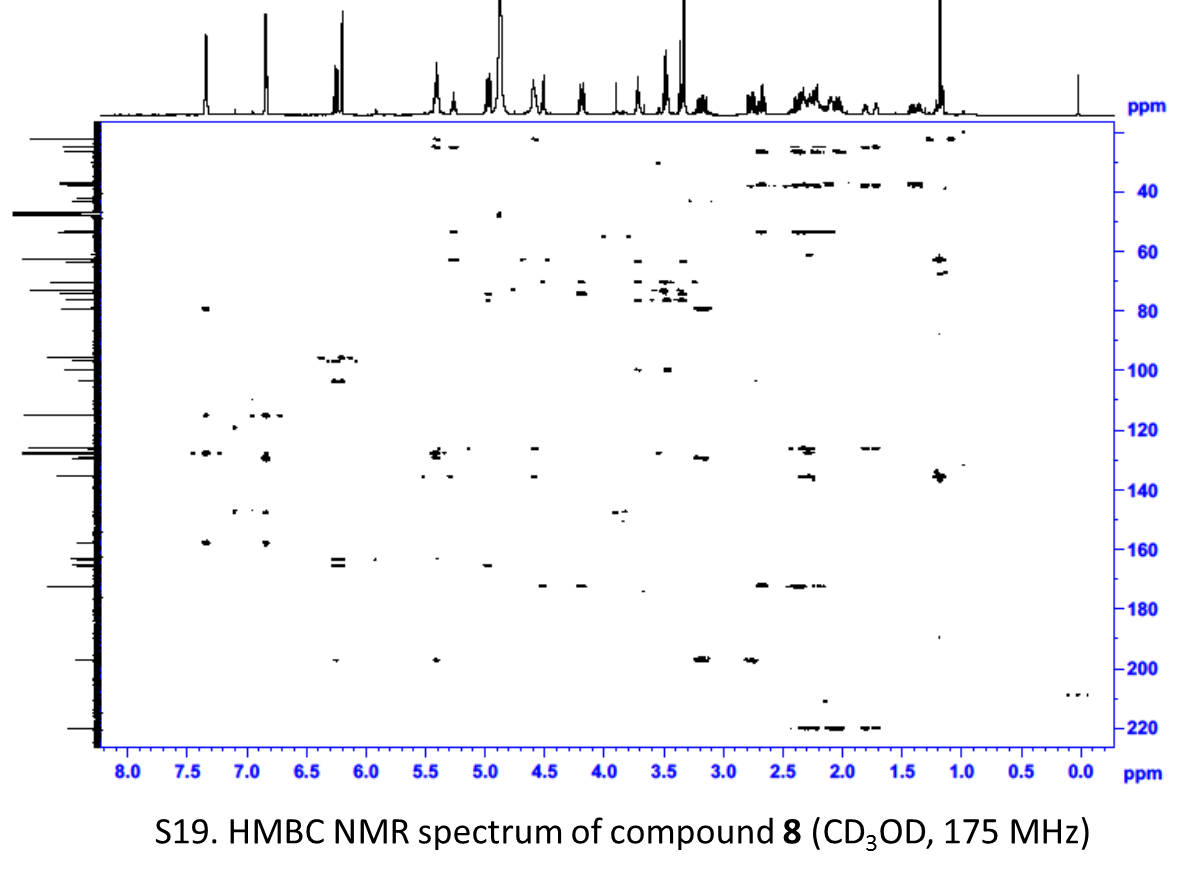


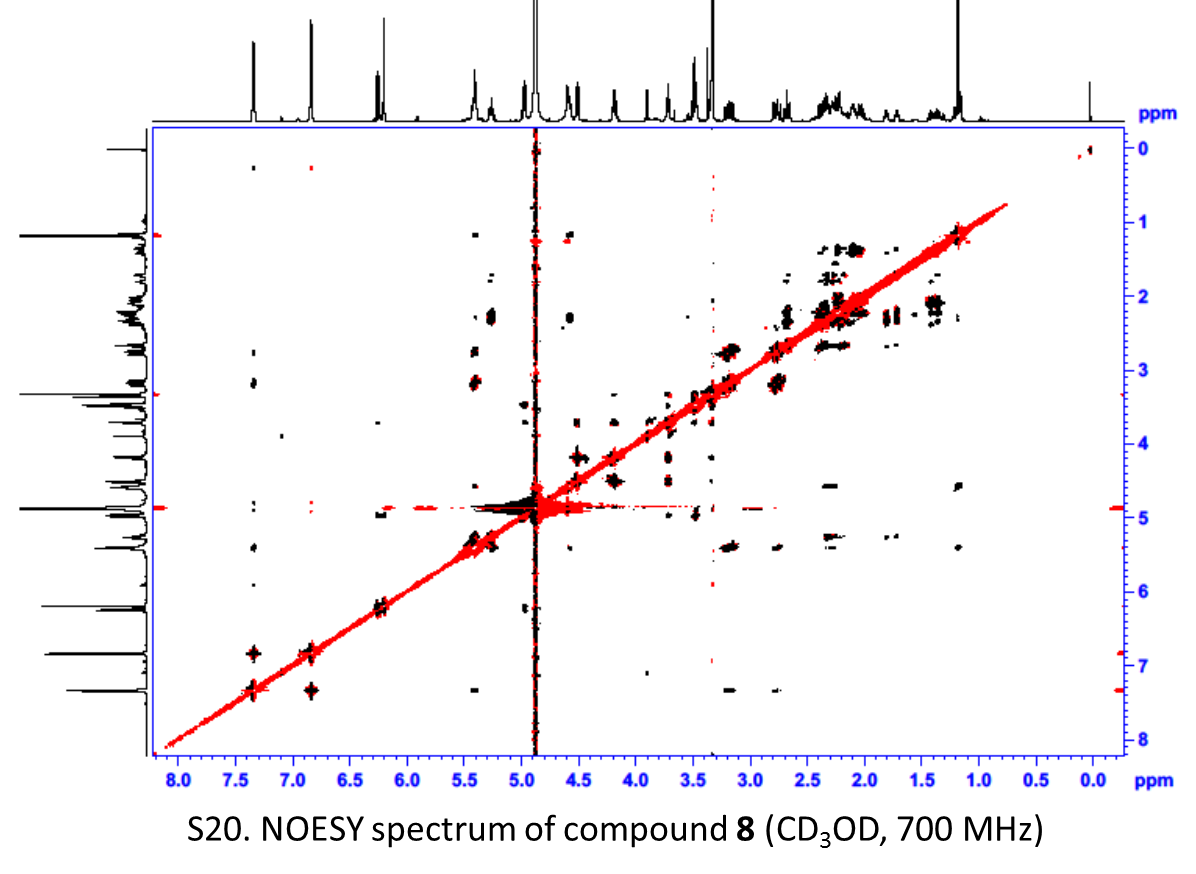


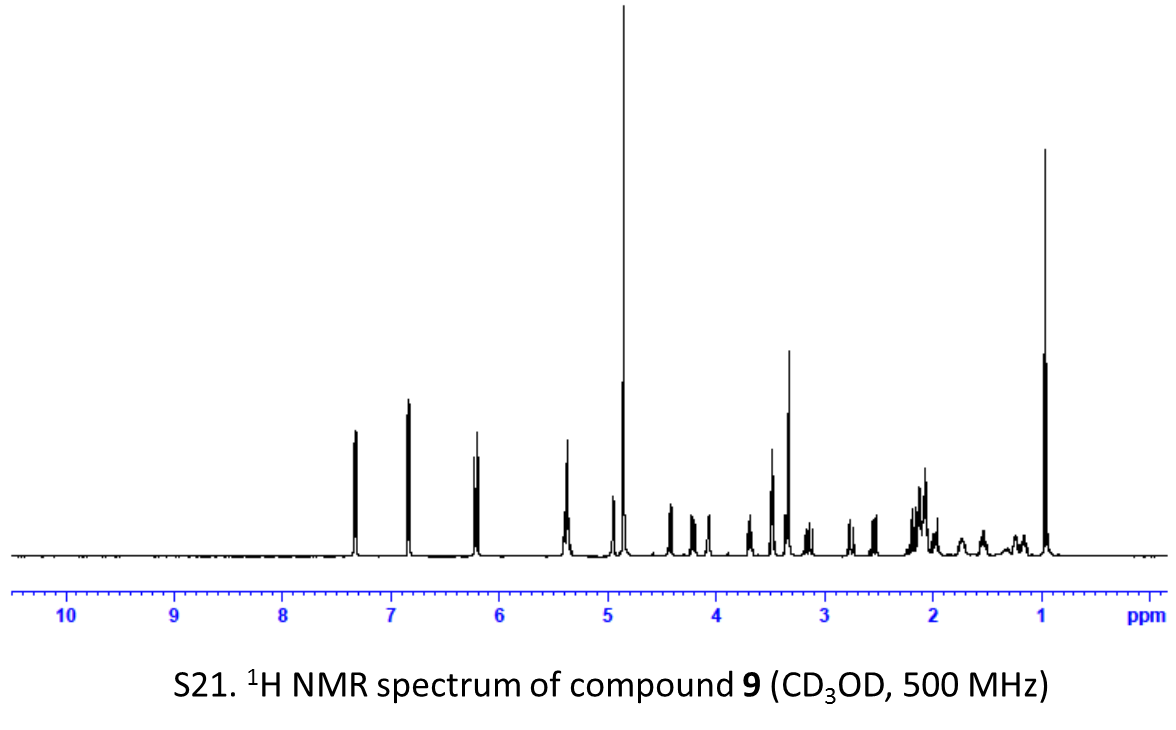


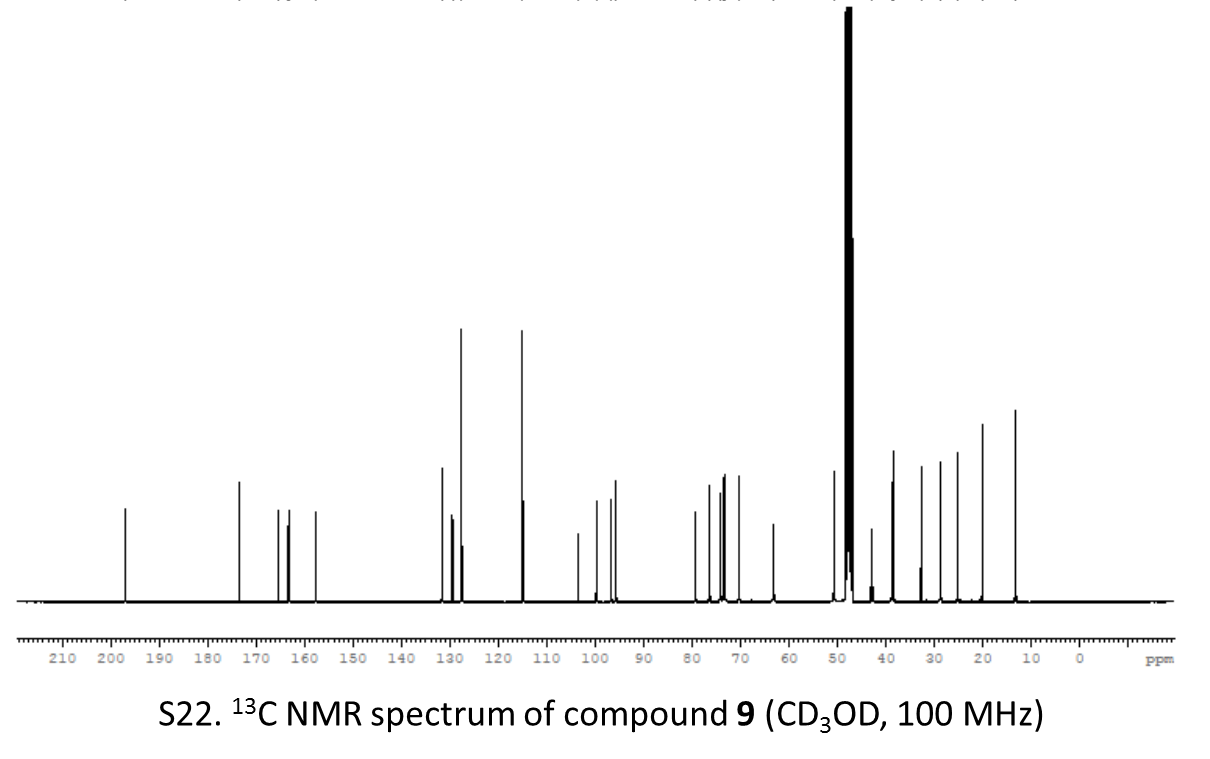


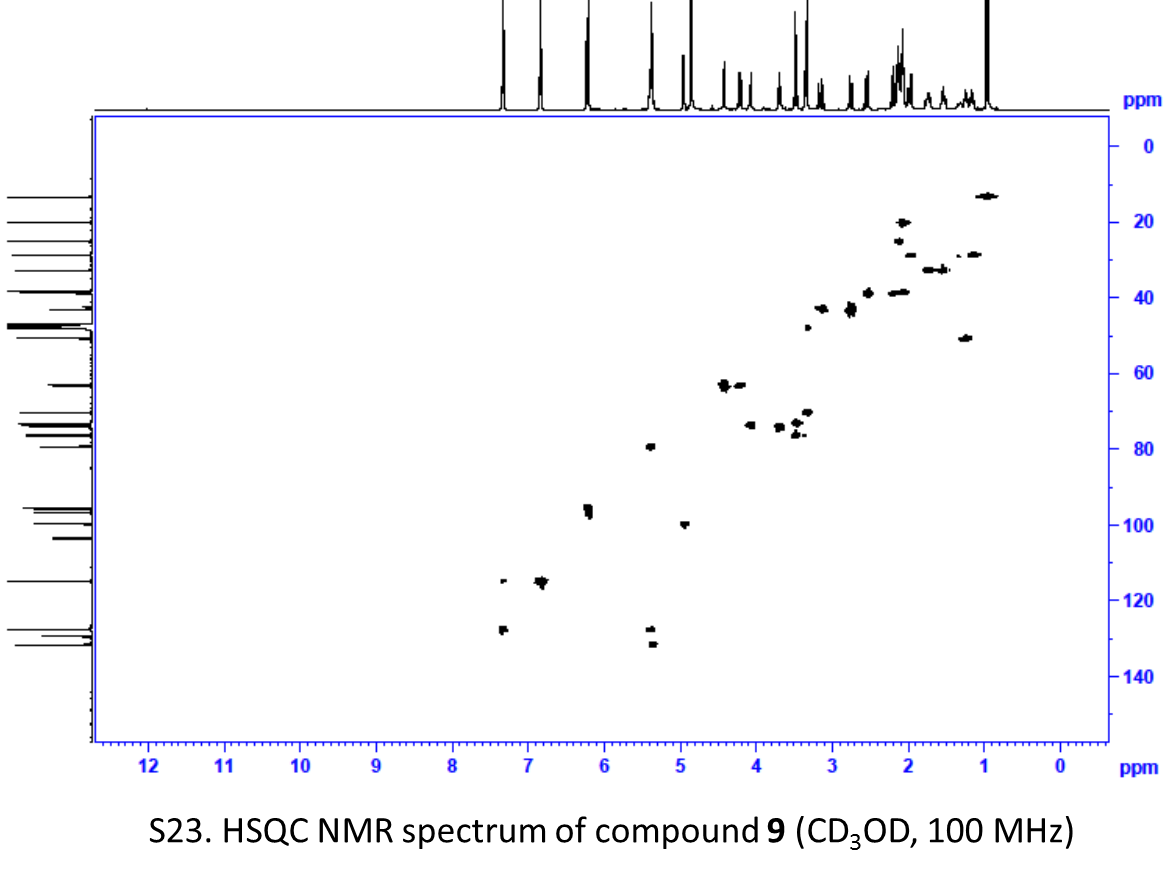

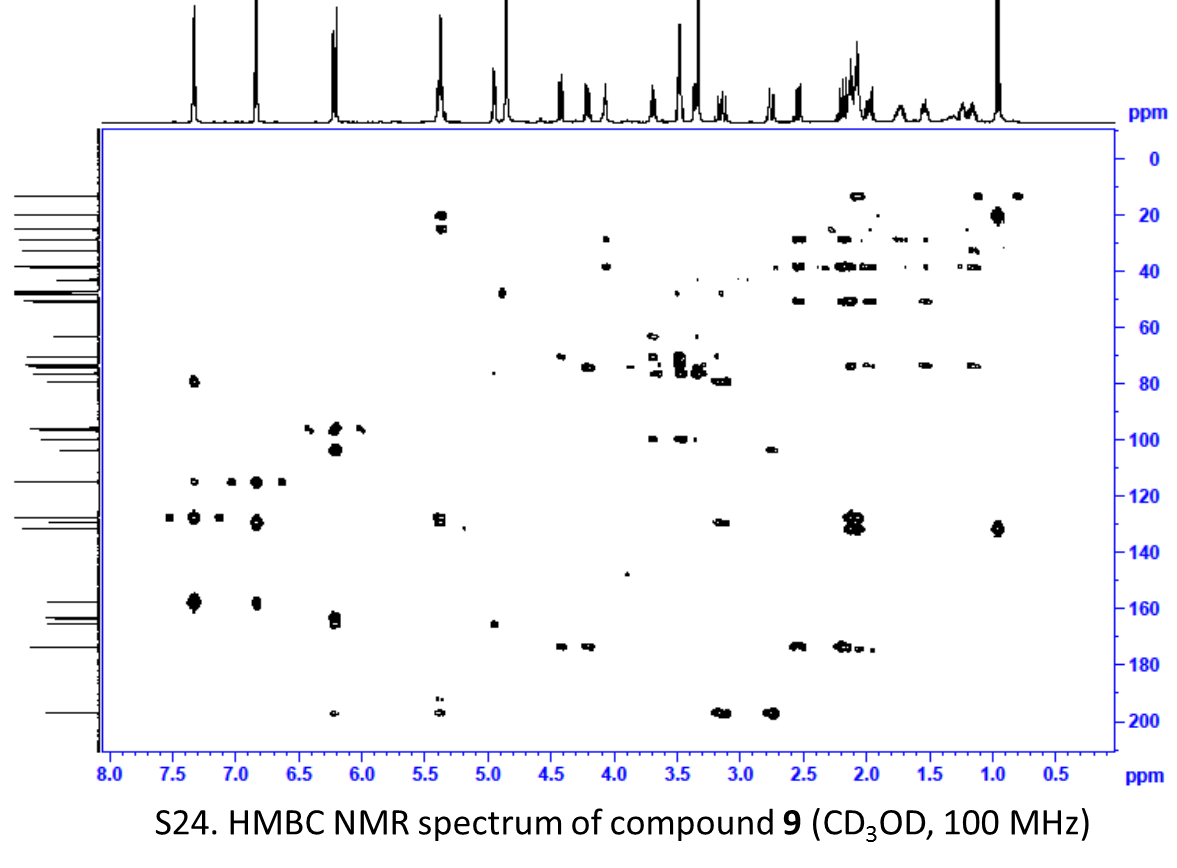


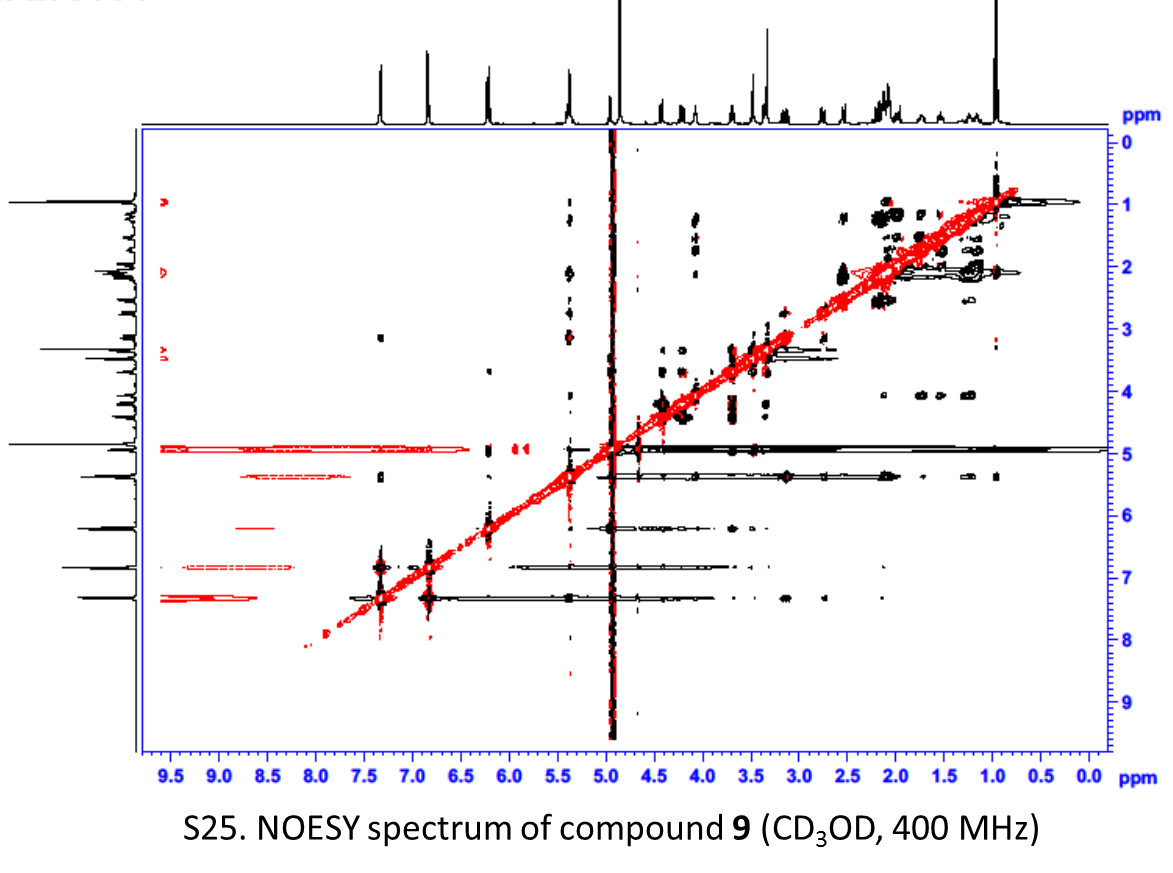


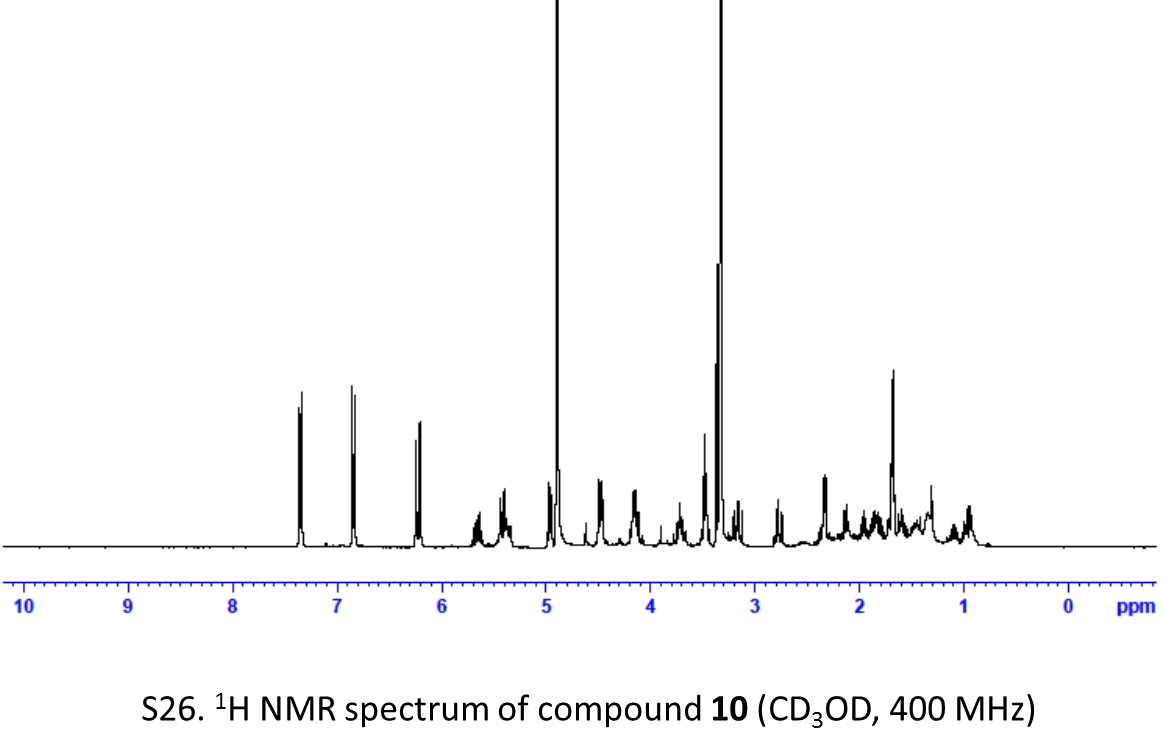


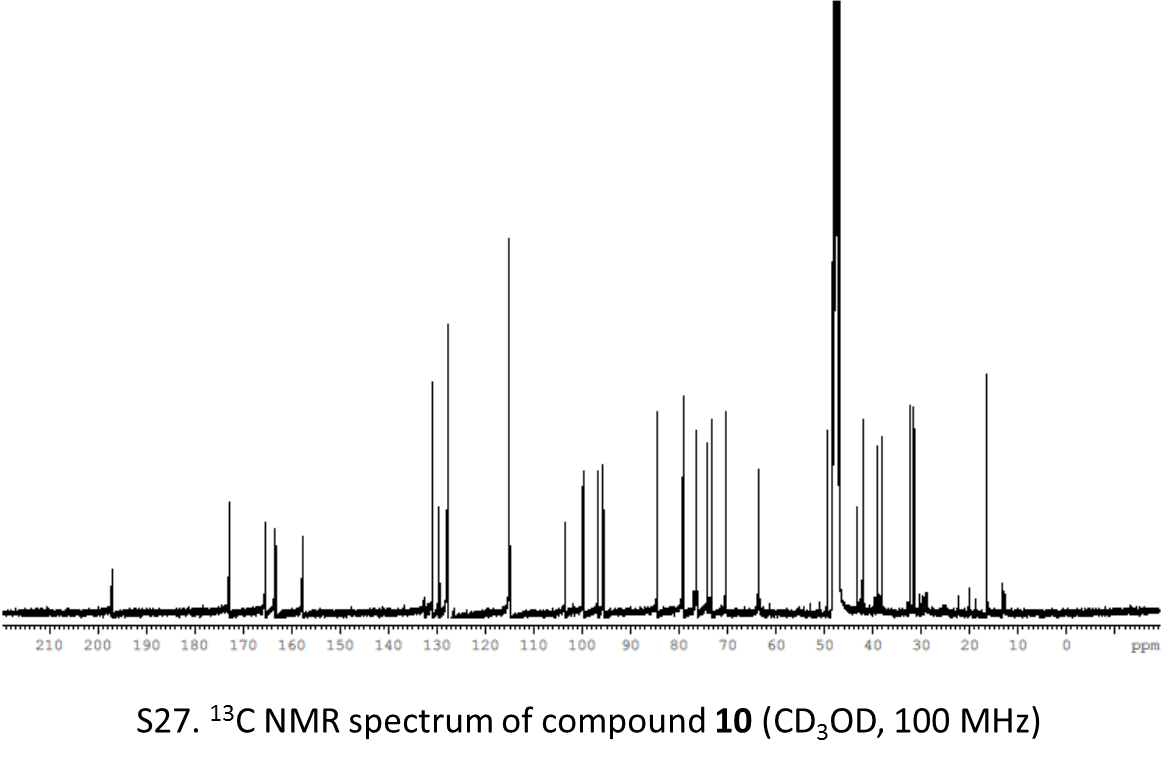


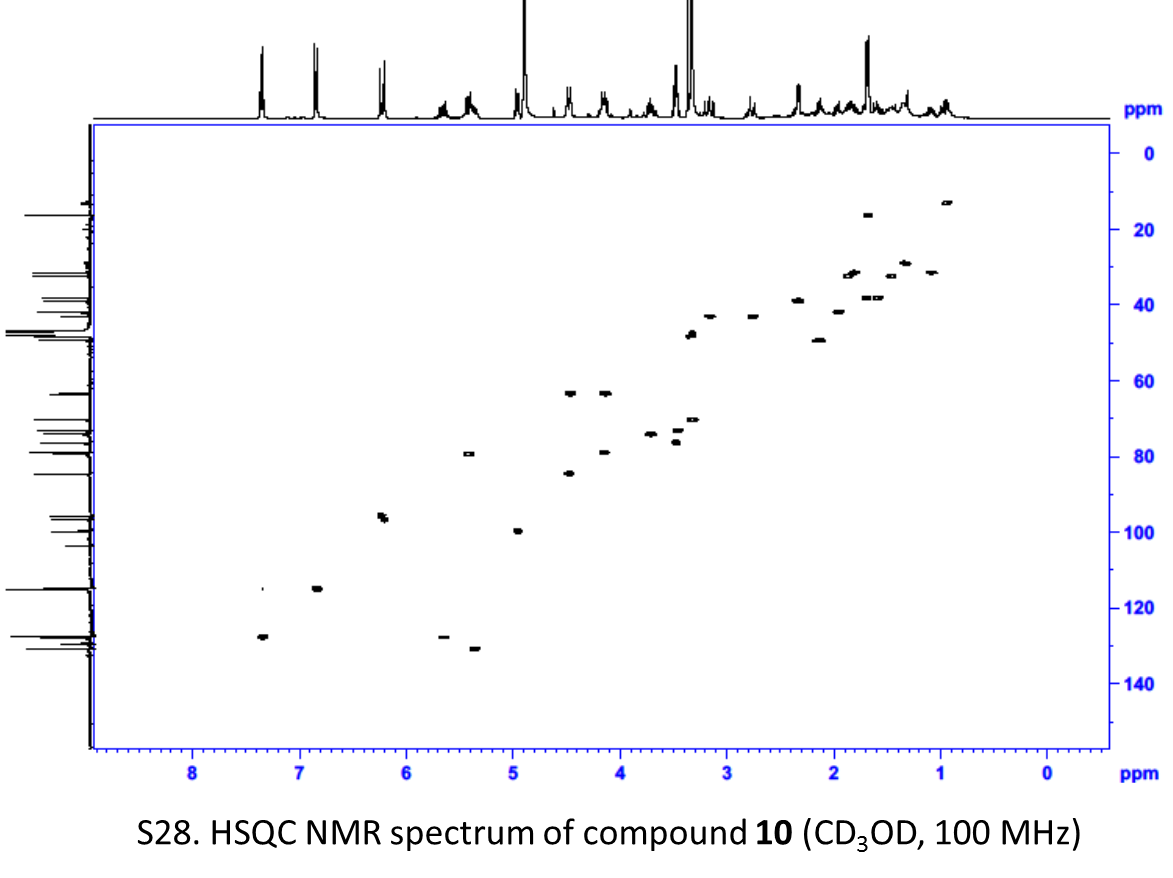


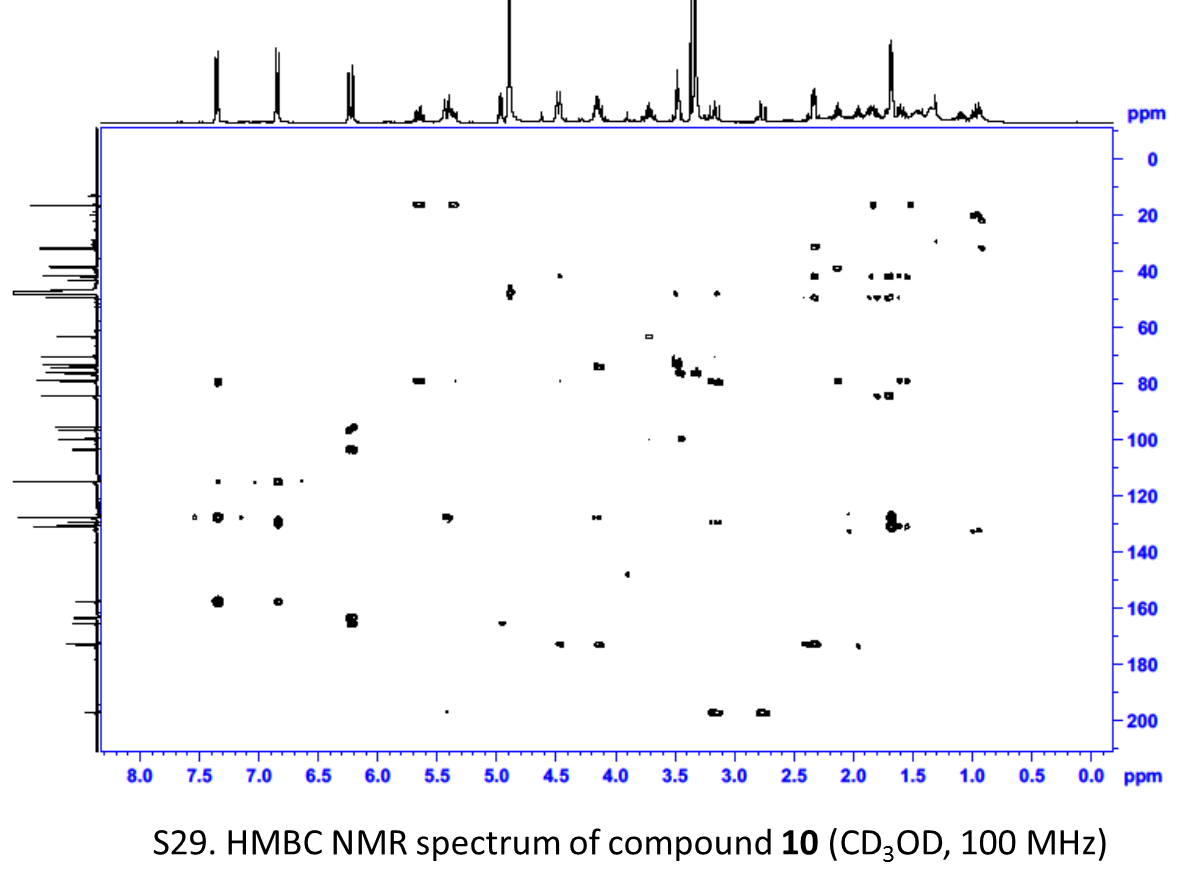


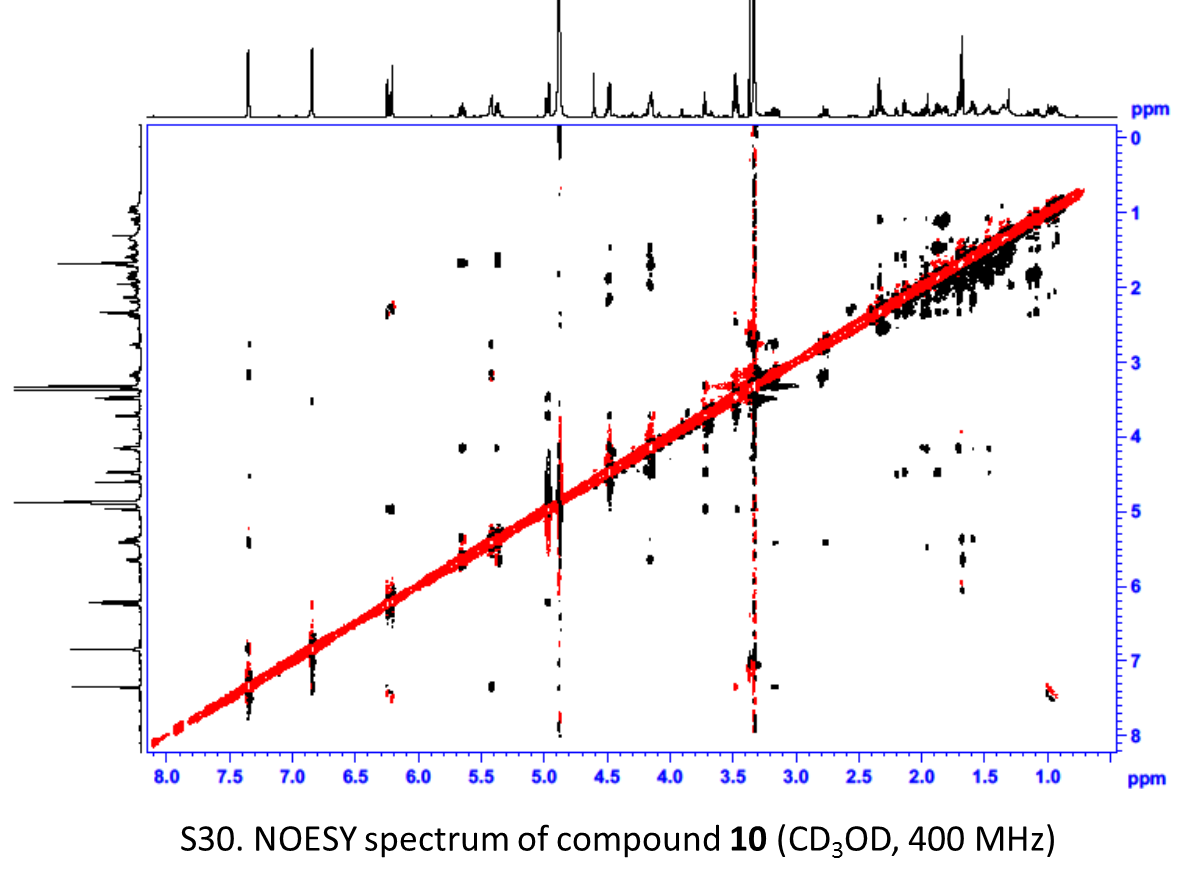


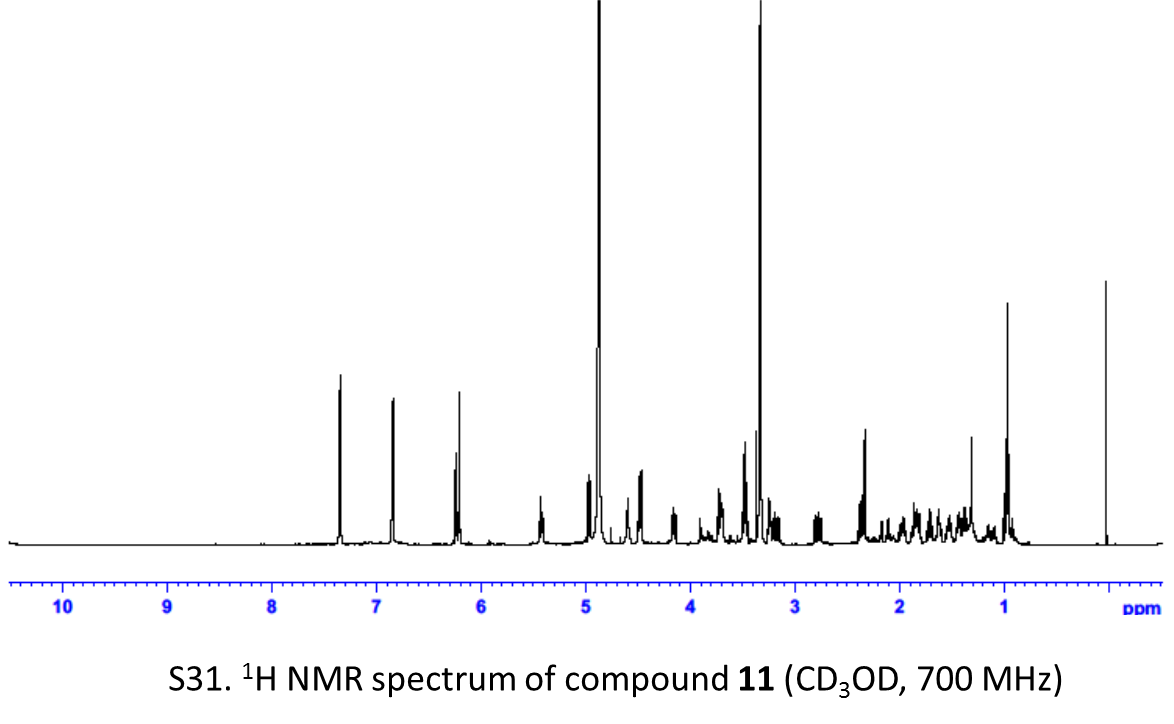


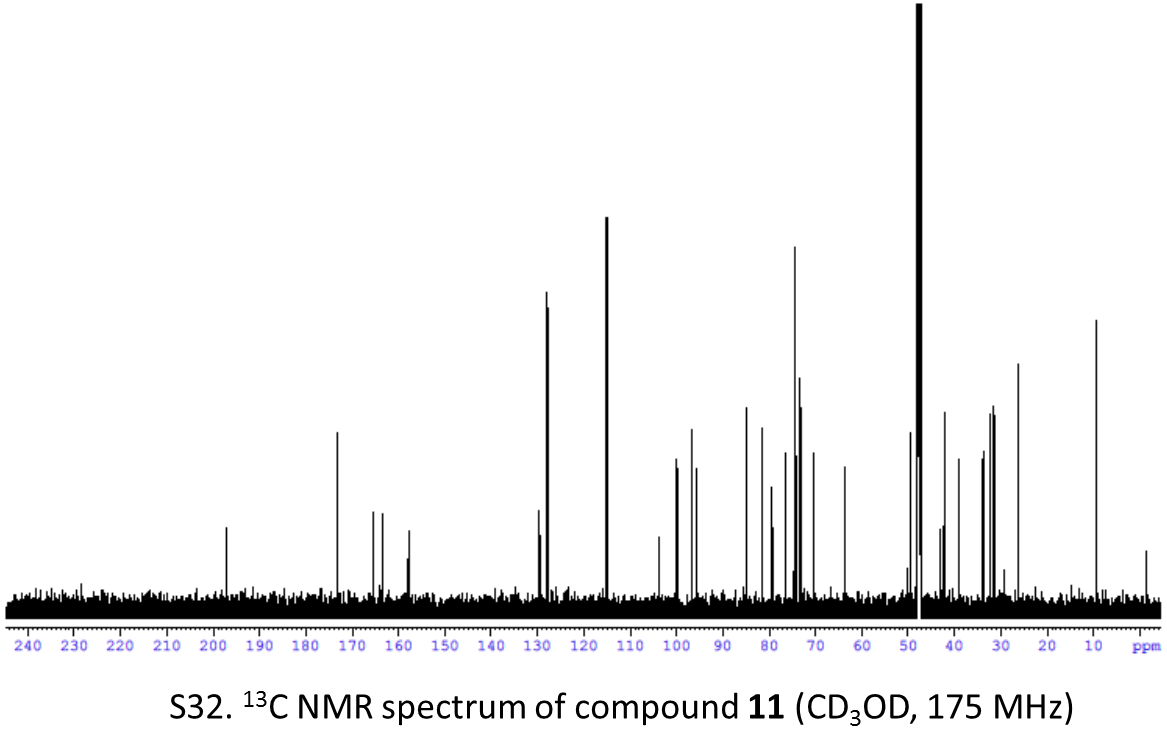


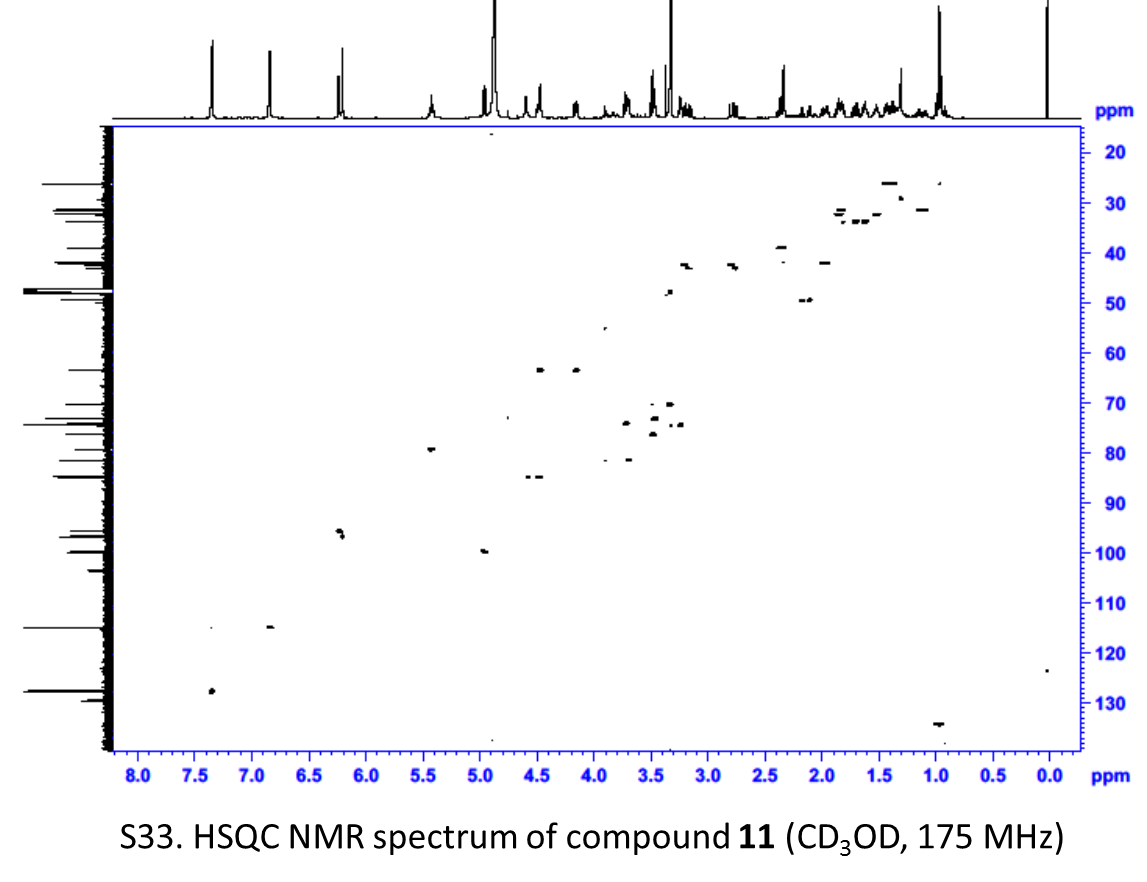

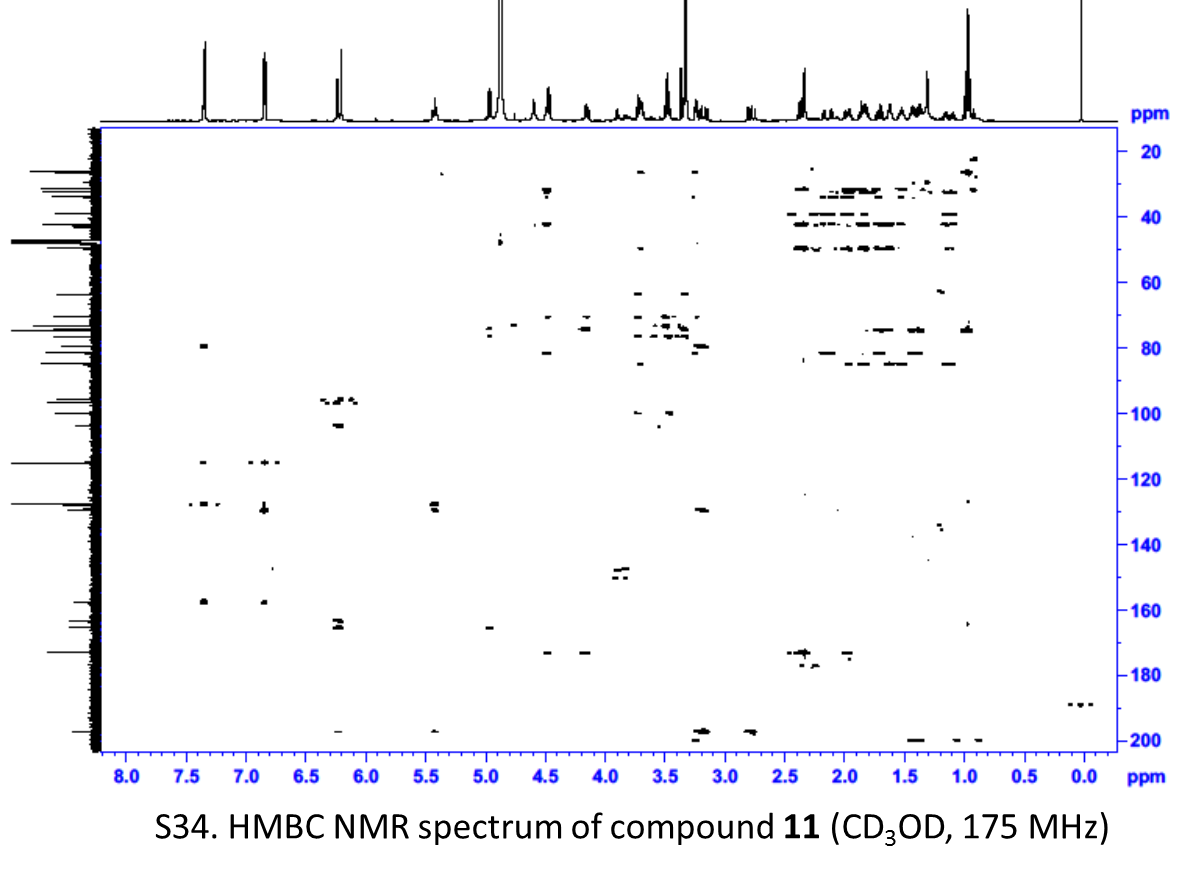


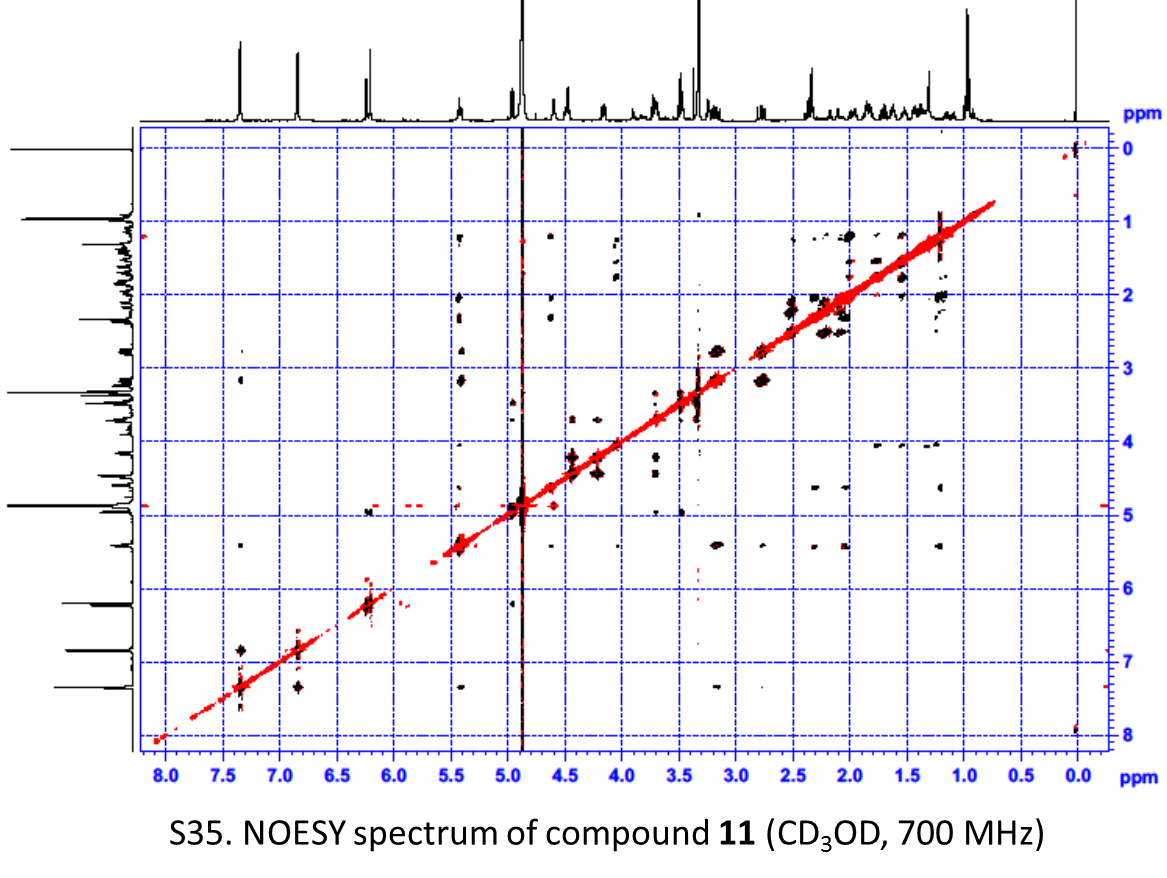

Supplement: Supplementary file 1 — Supplementary Information. [file 41598_2021_91850_MOESM1_ESM.docx]
